# Construction of a high-density genetic map and identification of QTLs related to agronomic and physiological traits in an interspecific (*Gossypium hirsutum* × *Gossypium barbadense*) F_2_ population

**DOI:** 10.1186/s12864-022-08528-2

**Published:** 2022-04-15

**Authors:** Zhanfeng Si, Shangkun Jin, Jiedan Chen, Sen Wang, Lei Fang, Xiefei Zhu, Tianzhen Zhang, Yan Hu

**Affiliations:** 1 Agronomy Department, College of Agriculture and Biotechnology, Zhejiang University, Hangzhou, 310029 China; 2 The Rural Development Academy Zhejiang University, Hangzhou, 310029 China; 3 State Key Laboratory of Crop Genetics and Germplasm Enhancement, Cotton Hybrid R & D Engineering Center (the Ministry of Education), College of Agriculture, Nanjing Agricultural University, 210095 Nanjing, China

**Keywords:** Cotton, Re-sequencing, Genetic map, QTL mapping, Agronomic and physiological traits

## Abstract

**Background:**

Advances in genome sequencing technology, particularly restriction-site associated DNA sequence (RAD-seq) and whole-genome resequencing, have greatly aided the construction of cotton interspecific genetic maps based on single nucleotide polymorphism (SNPs), Indels, and other types of markers. High-density genetic maps can improve accuracy of quantitative trait locus (QTL) mapping, narrow down location intervals, and facilitate identification of the candidate genes.

**Result:**

In this study, 249 individuals from an interspecific F_2_ population (TM-1 and Hai7124) were re-sequenced, yielding 6303 high-confidence bin markers spanning 5057.13 cM across 26 cotton chromosomes. A total of 3380 recombination hot regions RHRs were identified which unevenly distributed on the 26 chromosomes. Based on this map, 112 QTLs relating to agronomic and physiological traits from seedling to boll opening stage were identified, including 15 loci associated with 14 traits that contained genes harboring nonsynonymous SNPs. We analyzed the sequence and expression of these ten candidate genes and discovered that *GhRHD3* (*GH_D10G0500*) may affect fiber yield while *GhGPAT6* (*GH_D04G1426*) may affect photosynthesis efficiency.

**Conclusion:**

Our research illustrates the efficiency of constructing a genetic map using binmap and QTL mapping on the basis of a certain size of the early-generation population. High-density genetic map features high recombination exchanges in number and distribution. The QTLs and the candidate genes identified based on this high-density genetic map may provide important gene resources for the genetic improvement of cotton.

**Supplementary Information:**

The online version contains supplementary material available at 10.1186/s12864-022-08528-2.

## Background

*Gossypium hirsutum* and *G. barbadense* are the most two important cultivated species of allotetraploid cotton in the world. Among the domesticated *Gossypium* species, *G. hirsutum* is the most widely cultivated, dominating modern cotton production due to its high lint yield and broad adaptability [1, 2]; meanwhile, *G. barbadense* provides excellent fiber that is finer, longer and stronger than fiber of *G. hirsutum* [3, 4]. Efficient and extensive transmission of valuable genes between *G. barbadense* and *G. hirsutum* is of extremely important practical significance for improving fiber quality while maintaining fiber yield, which is mainly limited by linkage drag.

Quantitative traits exhibit continuous variation and are generally controlled by multiple genes, hence having a complex genetic basis; moreover, they are readily affected by the environment. Genetic research on quantitative traits is therefore difficult, and investigating the inheritance and QTL mapping of cotton quantitative traits is of great significance to the advancement of cotton genetics and breeding. Since Shappley et al*. *[5] constructed the first genetic map of cotton, many studies have conducted QTL mapping for important cotton traits.

A high-density molecular genetic map is the foundation of plant genome research. Interspecific maps have been constructed for cotton, mainly between *G. barbadense* and *G. hirsutum*, and used to explore species differences such as in yield and quality traits [6–27]. These studies have provided very useful information for cotton molecular design and breeding. There are many QTL-enriched regions in the cotton genome, and there may be large numbers of related genes that play important roles in the plant’s growth and development [28]. Notably, QTLs for important traits are unevenly distributed among 26 different chromosomes of cotton. In interspecific populations, fiber quality QTLs are more typically located in the A subgenome, while in intraspecific populations, fiber yield and quality QTLs are more frequent in the D subgenome [29, 30]. Although DD diploid species do not have spinnable fibers, many studies have shown that the D subgenome of allotetraploid cotton contains many QTLs that control fiber quality [31, 32]. However, while previous studies have revealed these and other useful findings, the different groups and markers employed combined with the impact of environmental factors on QTL effects mean that the comparability of extant data is relatively poor. Therefore, QTL research on cotton is still advancing.

Recent advances in genome sequencing technology allow the construction of ultra-high-density genetic maps based on SNP loci. Consequently, more comprehensive and accurate map information can be used to analyze QTLs associated with important traits. Since bin genetic linkage maps based on SNP loci were first constructed in rice [33], it has been widely applied in other plants such as cotton [20], maize [34], soybean [35], *Cucumis melo* [36], radish [37] and so on. These genetic linkage maps have yielded many fine-mapped QTLs for which corresponding target genes were identified and cloned.

Recent high-quality assemblies of *G. barbadense* and *G. hirsutum* [2, 38–42] has provided good references for linkage map-based QTL identification. In light of these resources, we constructed an interspecific F_2_ population between *G. hirsutum* and *G. barbadense* and performed whole-genome sequencing of all 249 F_2_ individuals, achieving resequencing data on average over 5 × genome coverage of each material and generating a high-density genetic map containing 6303 bin markers. Based on the map, we subsequently identified 112 QTLs associated with an array of traits including plant type traits and physiological traits at the seedling stage, leaf chlorophyll content, plant type traits at flower and boll stage, yield traits, and fiber quality traits. Combining the SNPs located within the predicted genes in the target region and their expression pattern of the predicted genes, possible causable genes that are responsible for the mapping traits were identified. These QTLs and the related candidate genes are valuable in cotton breeding to improve plant biomass, physiological characteristics, and yield quality.

## Methods

### Plant materials and DNA extraction

The plant materials used consisted of *G. hirsutum acc.* TM-1 supplied by Dr. Kohel of Southern Plain Agricultural Research Center, USDA [43] and *G. barbadense cv.* Hai7124 which was selected by Cotton Research Institute of Nanjing Agricultural University for genetic research [17]. TM-1 is a genetic standard line of *G. hirsutum* developed through single plant selection. Hai7124, grown extensively in China, was also the offspring of a single plant selection before being used as a parent in the construction of the linkage map. Two highly homozygous parents, as well as 249 F_2_ individuals derived from a cross between TM-1 as the recipient and Hai7124 as the donor were planted in Pailou greenhouse of Nanjing Agricultural University, Jiangsu, China. Genomic DNA was extracted from young leaf tissues following the method cetyltriethylammnonium bromide (CTAB) described by Paterson [44] with increased RNase A and proteinase K treatment to prevent RNA and protein contamination. The isolated DNA was then subjected to Illumina sequencing technology.

To obtain the phenotypic data of two parents, F_1_, and all 249 F_2_ individual plants at different environments. All of them were cut off the trunk, transferred in the large nutrient bowls, and moved into the greenhouse in autumn. In the next spring, these materials were planted in the field for investigation of yield and fiber traits. The same operation was repeated twice in 2011 and 2012.

## Phenotype data collection and evaluation

### Plant type traits at seedling stage

The following plant type traits of the parents, F_1_, and F_2_ individual plants respectively were investigated at the cotton seedling stage: plant height (PH1, cm); cotyledonary node height (CNH, cm); first true leaf height (FTLH, cm); second true leaf height (STLH, cm); distance between the cotyledonary node and first true leaf (D1, cm); and distance between first true leaf and second true leaf (D2, cm). Each measurement was repeated three times and the average value was used in the analysis.

### Physiological traits at seedling stage

Physiological characteristics such as leaf area and photosynthetic rate were measured in the parents, F_1_, and F_2_ individual plants at the cotton seedling stage. A portable leaf area meter (CI-202, Portable Laser Leaf Area Meter, USA) was used to measure the second true leaf area (SLA, cm^2^). At the same time, from 8:00 to 11:00 in the morning on a sunny day, a Li-6400 portable photosynthesis instrument was used to determine the photosynthesis ratio (Pn, μmol CO_2_·m^−2^·s^−1^) of the second true leaf. Also measured were intercellular CO_2_ concentration (Ci, μmol·mol^−1^), stomatal conductance (Cond, mmol·m^−2^·s^−1^), and transpiration rate (Tr, g·m^−2^·h^−1^). The intensity of the built-in light source was set to 1200 μmol·m^−2^·s^−1^, each leaf was measured three times, and the average value was used in the analysis. For instrument principle, sampling technique, and detailed settings, refer to "Using the LI-6400 Portable Photosynthesis System."

### Determination of chlorophyll content in leaves

The leaf chlorophyll content of the parents, F_1_, and F_2_ individual plants was determined by UV/visible spectrophotometer. The main stem functional leaves were collected from each individual plant, and ten pieces were cut out with a 9-mm punch and weighed. About 0.1–0.2 g leaves were then placed in a 10-ml test tube, the fresh weight recorded, 10 ml of 95% ethanol added, and the tube sealed and stored for 48 h in the dark. Tubes were shaken in the middle of the incubation and mixed until the leaves were completely white. After the incubation, the extracted chlorophyll of each sample was placed in a spectrophotometer and the optical density was measured at 665 nm, 649 nm, and 470 nm to respectively determine chlorophyll a (Chl a), chlorophyll b (Chl b), and carotenoid (Car) content. Subsequently, the chlorophyll a/b ratio (Chl a/b) and total chlorophyll (Total Chl) were calculated. Each sample was repeated three times, and the average was taken as the result.

Pigment concentrations were calculated according to the following formulas:

$$Ca\:=\:13.95D665-6.88D649$$

1 $$Cb\:=\:24.96D649-7.32D665$$

$$Cx\cdot c\:=\:(1000D470-2.05Ca-144.8Cb)/245$$

in which Ca, Cb, and Cx•c represent the concentration in mg/L of chlorophyll a, chlorophyll b and carotenoids, respectively.

The pigment content of the leaves was then calculated as follows:

$$Pigment\;content\;(mg/g)\:=\:CxV/1000\;W$$

where C represents the pigment concentration (mg/L), V represents the total amount of extract (ml), W represents the fresh weight of the sample (g), and the subscript x represents the pigment: chlorophyll a or b, or carotenoids.

### Plant type traits at flowering and boll stage

Plant height (PH2) and fruit branch number (FBN) were investigated at the first flowering and boll stage in the parents, F_1_, and F_2_ individual plants.

### Yield traits

Yield constituent factors were assayed during the boll opening stage. The traits investigated consisted of boll number per plant (bolls/plant, BN), seed cotton yield (SCY), lint yield (LY), boll weight (BW), lint percentage (LP), lint index (LI), and seed index (SI).

### Fiber quality traits

Middle and upper fibers were collected from the parents, F_1_, and F_2_ individual plants and sent for testing at the Cotton Quality Supervision, Inspection and Testing Center of the Ministry of Agriculture (HVI SPECTRUM 4.05.01 version, HVICC calibration level). Tested fiber quality properties included: fiber length (FL), fiber strength (FS), micronaire value (MIC), fiber length uniformity (FU), fiber elongation (FE). Due to high temperatures and too much rain in the summer of 2011, which caused abortion of pollen and super-separation of the sea-land hybrid population, some families failed to receive enough mature fiber, resulting in a lack of yield and quality trait data in some lines.

### Population DNA preparation, resequencing, and genotyping

Sequencing libraries were constructed with an insert size of 150 bp and sequenced on the Illumina HiSeq 2000 platform (Illumina, San Diego, CA, USA). To construct paired-end libraries, DNA was fragmented by sonication, and DNA ends were blunted before adding an A base to each 3′ end. DNA adaptors with a single T-base 3′ end overhang were ligated to the above products. Ligation products were purified on 2% agarose gels that each targeted a specific range of insert sizes. Quantification and quality assessment were carried out by running 1 μL of the library on an Agilent DNA 1000 LabChip analyzer (Agilent Technology 2100 Bioanalyzer). All raw reads were processed for quality control and filtered using fastp (https://github.com/OpenGene/fastp) with default parameters. The clean reads were mapped to the TM-1 reference genome [38] using Burrows–Wheeler Aligner (BWA) with the parameters of ‘mem -t 20 -M -R’. The mapping results were sorted and duplicates marked using functions implemented in SAMtools and Picard (http://broadinstitute.github.io/picard/). Only reads that mapped uniquely to the reference genome sequence were used to call SNPs. Identification of SNPs between the parental lines and F_2_ individuals was performed with Genome Analysis Toolkit 4 (GATK4). High-quality SNPs were filtered following the best practices workflow developed by the GATK team. SNPs with minor allele frequency (MAF) < 5% and represented in less than 30% of the F_2_ population were excluded using VCFtools. Polymorphic markers between the two parental lines were retained if they had the aa × bb segregation pattern in F_2_ individuals.

### Bin map construction

Recombinant breakpoints were identified using a slightly modified sliding window approach based on the ratio of SNP alleles derived from TM-1 and Hai7124 [38]. Consecutive 100-Kb intervals having the same genotype in the whole F_2_ population were merged as a recombination bin. Bins with significantly distorted segregation (*P*-value < 0.001) were filtered using the Chi-square test, and those remaining served as genetic markers for the construction of a genetic linkage map using Icimapping [45]. Collinearity between the genetic map and physical positions was visualized using ALLMAPS (https://github.com/tanghaibao/jcvi/wiki/ALLMAPS). A region containing three or more closely linked bins that exhibited significant segregation distortion (*P* < 0.001) was defined as an SDR.

### Statistics of phenotypic traits

For all traits, ANOVA was used to test for significant differences between parents, F_1_, and F_2_ individuals, and correlation coefficients and phenotypic variation were also calculated using SPSS v18.0 (SPSS, Chicago, IL, USA). The heterosis (H) of each trait is expressed by two values, mid-parent heterosis and over-parent heterosis: MH = (F_1_-MP)/MP × 100%, where MP is the average value of the parents.

### QTL mapping

IciMapping 3.0 (http://www.isbreeding.net) was used to detect the effects of QTLs in the F_2_ population. An LOD threshold of 2.5 was used to define significant additive QTLs; that is, when LOD ≥ 2.5 for a marker interval, it was considered to contain a significant QTL. At the same time, the additive effect (A), dominant effect (D), and contribution rate (R2) of each QTL on corresponding traits were calculated. The QTL genetic action mode uses the absolute value of D/A to judge the action effect of each QTL; a value greater than 1.20 indicates an over dominant effect, 0.81–1.20 a dominant effect, and 0.21–0.80 a partially dominant effect. Less than 0.20 indicates an additive effect. The method of naming QTLs follows that used for rice: QTL + traits + chromosome + QTL number.

### Candidate gene identification and expression

The putative candidate genes for the QTLs were predicted as follows. First, we analyzed the SNP types located in QTLs based on our assembled genome sequence for TM-1. We focused on significantly associated nonsynonymous SNPs located in exons or SNPs in the upstream of the candidate genes. Second, based on expression profiling data for sixteen vegetative and reproductive tissues from TM-1 (cotton.zju.edu.cn). We checked whether these selected candidate genes were dominantly and/or specific expressed in a development stage that is critical for the target trait. We further narrowed down the candidate genes according to their expression levels between TM-1 and Hai7124 (cotton.zju.edu.cn).

## Results

### High-density genetic map construction and characteristics of the bin marker loci

**Fig. 1:**
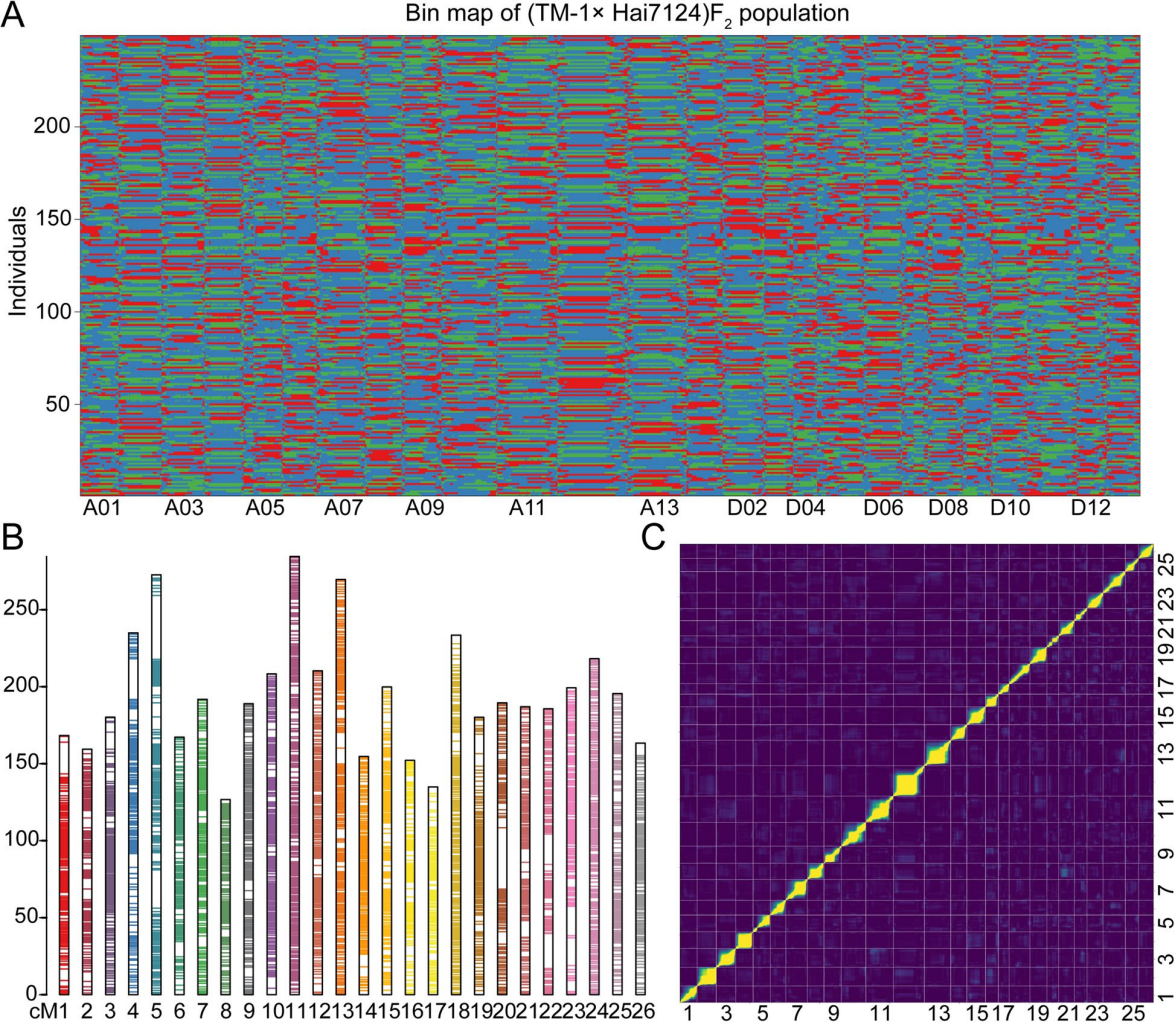
High-density genetic map construction of the (TM-1×Hai7124)F2 population. **A** Bin maps for the 241 scored F2 individual lines. Colored tracks represent the 241 individual lines of the THF2 population that were used for linkage map construction: red, alleles inherited from maternal parent (TM-1); green, alleles inherited from paternal parent (Hai7124); blue, alleles inherited from heterozygous genotype (TM-1 × Hai7124)F1. The horizontal scale indicates physical distance. **B** Distribution of markers across 26 chromosomes; ordinate is genetic distance, cM. **C** Genetic map quality as indicated by recombination fractions of all markers

We developed an interspecific F_2_ population from a cross between *G. hirsutum acc*. TM-1 and *G. barbadense cv*. Hai7124, which contained 249 individuals in total. Whole-genome sequencing of all individuals was performed on an Illumina Hiseq2000. In total, 3.01 Tb clean reads were generated, with an average of 5.3 × depth genome coverage for each individual. For the parents ‘TM-1’ and ‘Hai7124’, we utilized clean data from our previous research totaling 185 Gb and 111.8 Gb respectively [20], with an average depth of over 50 × . All clean reads were mapped to the TM-1 as the reference genome. After filtering SNPs by established criteria, a total of 4,257,943 high-quality SNPs (Fig. 1) were retained and used to generate bin markers (a group of consecutive SNPs in the same block for genotyping) with a modified sliding window approach [33]. After filtering 1428 bins that exhibited significant segregation distortion (*P* < 0.001), a total of 6303 bin markers were generated, with an average length of 363.1 Kb (Table 1, Fig. 1). Finally, the high-density genetic map was constructed, covering 5057 cM with an average inter-bin genetic distance of 0.8 cM (Fig. 1, Table 1). The 26 linkage groups of the map was corresponding to 26 cotton chromosomes. Each of the linkage group contained 242.4 bins on average, ranging from 157 (D04) to 405 (A11), overall comprising 3,455 in the A subgenome and 2,848 in the D subgenome. The total length of the A subgenome was 2663.24 cM, and that for the D subgenome was 2393.89 cM. The longest linkage group was A11 of 284.58 cM, and the shortest one was A08 of 126.68 cM. The largest average distance between markers was 1.1 cM in the D07 linkage group, while the smallest average distance between markers in A10 was 0.64 cM (Table 1).

**Table 1:** Characteristics of the 26 linkage groups in allotetraploid cotton

Chr	No. bins	Distance (cM)	Average distance	Average length of bin (Kb)	Gap number (> 10 cM)	Recombination rate (cM/Mb)	RHRs
A01	234	168.32	0.72	505.0	1	1.42	104
A02	197	159.41	0.81	549.6	0	1.47	76
A03	254	180.2	0.71	439.3	1	1.61	113
A04	239	234.94	0.98	366.9	1	2.68	110
A05	256	272.68	1.07	433.0	2	2.46	147
A06	206	167.24	0.81	614.0	0	1.32	99
A07	255	191.84	0.75	378.8	0	1.99	138
A08	189	126.69	0.67	661.7	0	1.01	78
A09	262	189.01	0.72	317.6	0	2.27	150
A10	326	208.36	0.64	353.1	0	1.81	151
A11	405	284.58	0.7	299.7	0	2.34	219
A12	284	210.33	0.74	378.8	0	1.95	166
A13	348	269.64	0.77	317.1	2	2.44	166
At-Total	3455	2663.24	0.78	431.89	7	1.78	1717
D01	232	154.67	0.67	278.9	0	2.39	119
D02	257	199.83	0.78	271.5	0	2.86	115
D03	173	152.18	0.88	311.5	0	2.82	91
D04	157	134.93	0.86	362.6	0	2.37	95
D05	282	233.47	0.83	226.7	0	3.65	207
D06	221	180.13	0.82	296.2	1	2.75	103
D07	173	189.57	1.10	337.7	1	3.25	107
D08	217	187.01	0.86	318.3	1	2.71	127
D09	185	185.67	1.00	281.1	2	3.57	121
D10	228	199.29	0.87	293.3	2	2.98	126
D11	317	218.19	0.69	225.1	0	3.06	200
D12	195	195.58	1.00	316.4	0	3.17	143
D13	211	163.37	0.77	305.4	0	2.53	109
Dt-Total	2848	2393.89	0.86	294.21	7	2.85	1663
Total	6303	5057.13	0.80	363.1	14	2.21	3380

A total of fourteen gaps that larger than 10 cM were distributed across the all 26 chromosomes, seven at the A subgenome and seven at the D subgenome. The average ratio of bin marker interval (< 5 cM) for all linkage groups was more than 99%. A region containing three or more closely linked bins that exhibited significant segregation distortion (*P* < 0.001) was considered a segregation distortion region (SDR). There were 88 and 32 SDRs in the A and D subgenome, respectively (Table 1). The quality of the genetic map was further examined by comparing genetic and physical distances, which showed good collinearity (Supplementary Fig. 1).

Chi-square tests of the 6303 co-dominance bins identified 724 that do not conform to the 1:2:1 genetic law ratio of Mendelian theory. Among these 724 partial segregation bins, 86 were biased toward the parent TM-1, 638 toward the parent Hai7124, and none toward the heterozygote. In addition, significantly more of the partial segregation bins were located on the A subgenome (450) than on the D subgenome (274), and these bins comprised a higher proportion of the A subgenome (13.02%) than of the D subgenome (9.62%). Moreover, the partial segregation bins were unevenly distributed across the 26 chromosomes; the ratio of partial segregation bins to total bins in a given chromosome was more than 30% on chromosomes A05, A11, and D07 and more than 20% on A08, D09, and D10, but less than 1% on A01, A03, and D01. At the same time, some partial segregation bins exhibited an aggregation phenomenon; namely, bins distributed on four chromosomes (A05, A11, D07, and D08) account for 45% of all partial segregation bins (Supplementary Table 1).

**Fig. 2:**
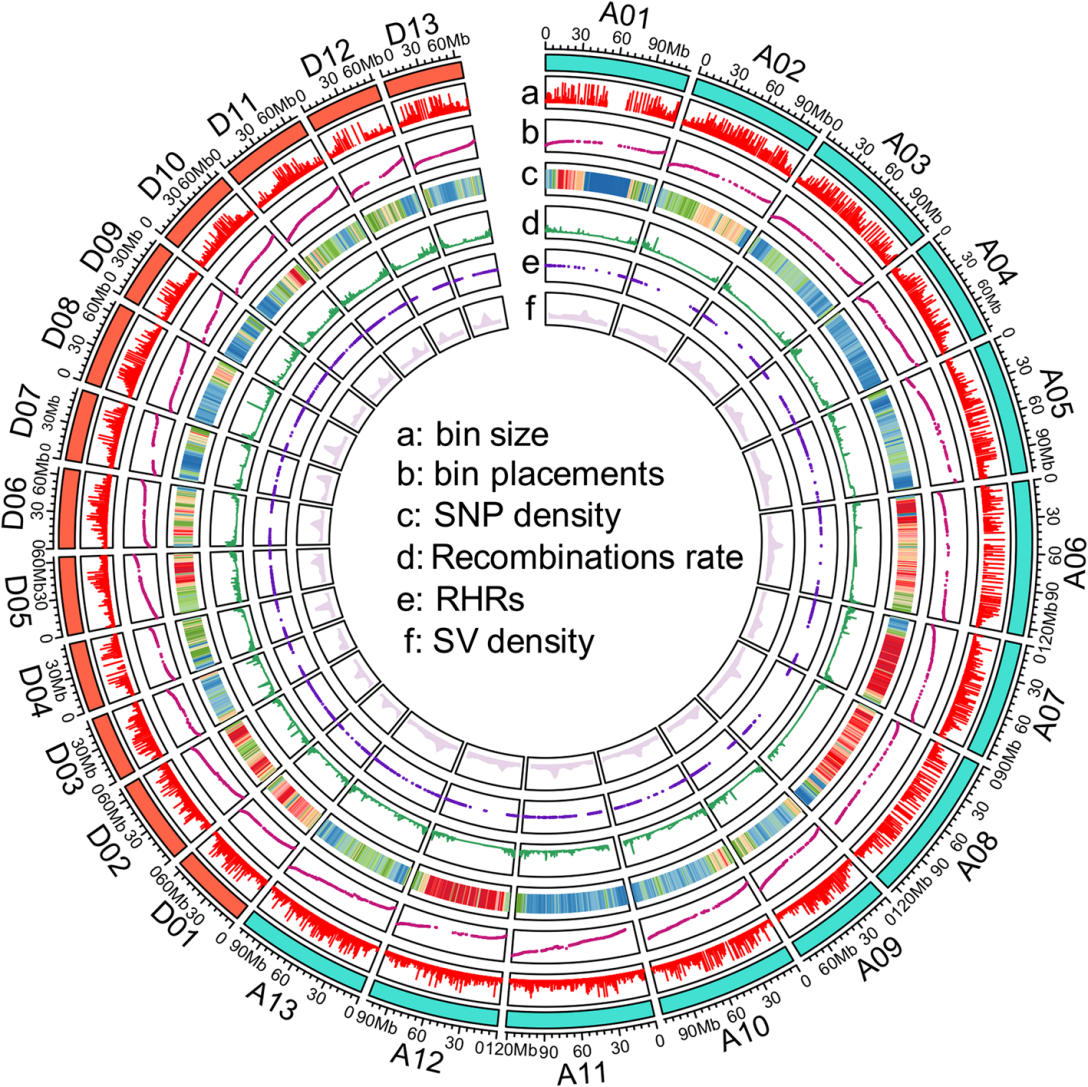
Chromosomal features of (TM-1 × Hai7124)F_2_ population with genetic data. **A** The length of 6303 bins along each chromosome; **B** The bin marker placements in the genetic maps on the chromosome; **C** SNP (aa × bb) density of each chromosome; **D** Recombination rates of each chromosome; **E** Genetic positions of the RHRs in each chromosome; **F** Structural variations density of each chromosome

To provide a comprehensive overview of recombination in cotton, the recombination rate along each chromosome was estimated by comparing genetic and physical distances. Across the entire genome, the average recombination rate was 2.2 cM/Mb. High rates of recombination were observed in the telomere regions of all nine chromosomes, whereas recombination was suppressed in centromere regions (Fig. 2). Chromosomal regions with recombination rates greater than 1.0 cM/Mb [37] were defined as recombination hot regions (RHRs). A total of 3380 RHRs were identified, and were unevenly distributed on the 26 chromosomes (Table 1, Fig. 2).

### Analysis of 35 traits in parents and F_1_ and F_2_ generations

We surveyed 35 traits in the parents and F_1_ and F_2_ generations, including six plant type traits, ten leaf morphology and physiological traits at the seedling stage, five leaf chlorophyll content traits, two plant type traits at flower and boll stage, seven yield traits, and five fiber quality traits (Supplementary Table 2).

TM-1, Hai7124, and their F_1_ progeny differed to varying degrees in plant type, leaf morphology, physiology, yield, and fiber quality. Concerning plant type traits, TM-1 and F_1_ had extremely significant differences; TM-1 and Hai7124 likewise had extremely significant differences, except in CNH; but Hai7124 and F_1_ had no extremely significant differences in traits except for D1. Regarding leaf morphology and physiological traits, TM-1 exhibited extremely significant difference from Hai7124 and from F_1_ only in SLA and SPn; other traits were not significantly different among the three. In terms of chlorophyll content, TM-1 and F_1_ exhibited extremely significant differences; TM-1 and Hai7124 likewise had extremely significant differences in traits other than Chla; but Hai7124 and F_1_ did not differ significantly except in Chla/b. With regard to the 12 yield and fiber traits, *G. hirsutum* and *G. barbadense* are characterized by extremely significant differences; most of these characteristic differences were observed in comparisons of TM-1 and Hai7124 and of TM-1 and F_1_ individuals. When comparing Hai7124 and F_1_, only the five traits D1, SCY, LY, SI, and LI differed significantly, indicating that the F_1_ progeny of *G. barbadense* and *G. hirsutum* are more biased towards the *G. barbadense* phenotype. Taken together, these genetic differences provide a good basis for the screening of important trait QTLs (Supplementary Table 3).

In the F_2_ population, the average value and variance of each trait exhibited large changes relative to their parents, and the coefficients of variation differed between traits. Overall, physiology and yield traits featured the largest coefficients; the values for each yield component ranked as follows: BN > SCY > LY > LI > BW > SI > LP. This ranking indicates that in the offspring, different degrees of genetic variation are present for different traits, indicating that these traits are controlled by multiple genes (Table 2, Supplementary Fig. 2).

**Table 2:** Phenotypic variation of 35 traits

**Traits**	**Number of individuals**	**Mean**	**Mean squared error**	**Min**	**Max**	**Standard deviation**	**variance**	**Skewness**	**kurtosis**	**CV (%)**	**mid-parent heterosis**
PH1	249	20.4	0.23	9.30	32.30	3.69	13.61	-0.41	0.14	18.08	-4.23
CNH	249	7.59	0.08	3.50	11.40	1.32	1.75	0.11	0.28	17.41	9.21
FTLH	249	15.42	0.17	8.20	23.00	2.73	7.47	0.11	-0.2	17.73	6.53
STLH	247	18.62	0.2	8.90	30.50	3.14	9.84	-0.2	0.48	16.85	-2.10
D1	249	7.83	0.14	1.90	14.10	2.15	4.63	0.36	0.09	27.49	4.05
D2	247	3.19	0.11	0.20	9.80	1.79	3.21	0.64	0.25	56.12	-29.89
SLA	249	30.6	0.54	7.93	61.66	8.54	72.87	0.43	0.68	27.90	4.42
TLA	233	25.07	0.64	4.99	54.89	9.80	96.08	0.34	-0.19	39.10	-3.00
SPn	249	9.24	0.25	0.74	18.64	3.95	15.64	0.05	-0.71	42.79	6.39
TPn	249	11.62	0.25	3.24	20.89	3.98	15.85	-0.18	-0.79	34.27	1.18
SCi	249	235.77	3.14	84.24	325.24	49.53	2452.76	-0.64	0.10	21.01	1.34
TCi	249	237.19	3.13	30.12	369.80	49.37	2437.74	-0.59	0.78	20.82	3.63
SCond	249	0.13	0.01	0.01	0.37	0.08	0.01	0.56	-0.51	63.78	23.81
TCond	249	0.16	0.01	0.02	0.44	0.09	0.01	0.42	-0.5	54.37	14.29
STr	249	4.64	0.19	0.49	12.90	2.92	8.54	0.63	-0.56	63.02	16.88
TTr	249	5.53	0.18	0.71	12.83	2.89	8.38	0.59	-0.44	52.34	10.82
Chl a	240	0.78	0.01	0.37	1.27	0.13	0.02	0.30	1.49	16.42	4.00
Chl b	240	0.28	0.00	0.15	0.44	0.04	0.00	0.34	0.96	15.69	12.00
Car	240	0.16	0.00	0.07	0.27	0.03	0.00	0.25	1.19	17.30	-5.88
Chl a/b	240	2.82	0.01	2.04	3.21	0.17	0.03	-0.42	1.37	6.06	-6.16
Total Chl	240	1.06	0.01	0.53	1.71	0.17	0.03	0.32	1.39	16.02	6.00
PH2	238	144.01	2.26	48.00	220.00	34.94	1220.57	-0.66	0.41	24.26	48.21
FBN	238	13.08	0.26	3.00	24.00	4.06	16.50	-0.37	-0.04	31.05	-12.80
BN	224	13.62	0.52	0.00	42.00	7.84	61.40	1.14	1.13	57.55	-34.09
SCY	215	48.81	1.87	4.08	142.83	27.45	753.45	1.00	0.67	56.24	-36.10
LY	215	14.72	0.55	1.11	40.32	8.08	65.36	0.89	0.34	54.93	-41.27
BW	220	3.56	0.06	1.57	7.47	0.92	0.85	0.75	1.18	25.93	-13.59
SI	220	10.45	0.13	5.85	20.00	1.99	3.96	0.67	2.02	19.04	-3.86
LP	220	30.65	0.36	20.79	52.38	5.38	28.98	0.97	1.63	17.56	-7.49
LI	220	4.64	0.08	2.27	11.14	1.26	1.58	1.39	4.87	27.09	-13.91
FL	77	29.69	0.24	25.41	35.43	2.06	4.26	0.21	-0.09	6.95	-3.49
FS	77	30.26	0.34	25.00	40.90	3.02	9.13	0.89	1.35	9.98	-5.19
MIC	77	3.14	0.07	2.00	4.79	0.63	0.40	0.23	-0.31	20.03	-28.15
FU	77	83.9	0.21	78.8	88.20	1.81	3.29	-0.32	0.19	2.16	-1.53
FE	77	6.26	0.05	5.20	7.98	0.47	0.22	1.31	2.84	7.43	-5.22

### QTL mapping of important agronomic traits in cotton

A total of 112 QTLs, 41 in the A subgenome and 71 in the D subgenome, distributing across almost all 26 chromosomes except A03, A08, and D08, were assessed for association with 35 traits using ICIM analysis. The position, LOD score, additive effects, dominance effect, and percentage of phenotypic variance explained (PVE) of the QTLs are given in Table 3. Among them, 16 QTLs were located overlapped with the QTL regions in the previous studies (Table 4). PVE values ranged from 2.95 to 24.89%. The regions occupied by identified QTLs ranged in size from 0.20 to 8.45 Mb, with an average length of 0.78 Mb. With respect to traits, the number of QTLs per trait ranged from 0 to 10 with the most QTLs (up to 10) being detected for STr.

**Table 3:** Analysis of QTLs for 35 traits

Trait	QTL	Chromsome	Left Marker	Right Marker	LeftCI (cM)	RightCI (cM)	Start	End	LOD	PVE(%)	Add	Dom
PH1	*qPH1-A13*	A13	bin395	bin396	248.5	252.5	107,859,745	108,097,137	3.16	6.89	-1.09	-0.8
PH1	*qPH1-D05*	D05	bin223	bin229	155.5	167.5	39,478,283	41,627,412	3	6.47	-1.21	0.7
CNH	*qCNH-A07*	A07	bin3	bin4	0	6.5	330,907	915,621	3.37	6.65	-0.32	0.51
CNH	*qCNH-A12*	A12	bin454	bin455	181.5	186.5	104,326,658	104,861,614	3.95	8.35	0.5	-0.1
FTLH	*qFTLH-A04*	A04	bin2	bin3	2.5	6.5	295,380	912,894	2.98	6.3	-0.49	-1.1
STLH	*qSTLH-A13*	A13	bin393	bin394	245.5	248.5	107,403,460	107,859,745	3.02	4.86	-0.98	-0.25
STLH	*qSTLH-D01*	D01	bin57	bin58	51.5	54.5	10,754,698	11,063,172	3.04	4.93	1.09	-0.06
STLH	*qSTLH-D05-1*	D05	bin221	bin227	151.5	154.5	52,507,478	52,707,478	2.98	4.55	-0.55	1.2
STLH	*qSTLH-D05-2*	D05	bin250	bin251	173.5	174.5	38,370,762	40,442,855	3.22	5.01	-0.63	1.21
D1	*qD1-A01*	A01	bin58	bin59	53.5	55.5	12,558,163	13,053,104	2.75	5.62	0.68	0.13
D1	*qD1-A04*	A04	bin2	bin3	1.5	5.5	295,380	912,894	3.44	7.18	-0.25	-1.02
D2	*qD2-A11*	A11	bin7	bin55	29.5	48.5	807,428	9,258,460	3.68	4.46	-1.3	-1.28
SLA	*qSLA-A09*	A09	bin176	bin177	103.5	105.5	63,066,211	63,735,059	2.62	7.16	0.38	3.73
SPn	*qSPn-D05*	D05	bin59	bin60	43.5	44.5	8,567,785	8,789,652	4.35	7.56	1.6	0.01
SPn	*qSPn-D06*	D06	bin6	bin7	0	1.5	617,114	818,140	2.87	4.89	0.28	-1.78
SPn	*qSPn-D12*	D12	bin138	bin139	107.5	110.5	47,167,180	47,756,249	3.31	5.69	-0.01	1.87
SPn	*qSPn-D13*	D13	bin209	bin211	135.5	139.5	61,357,809	61,953,285	3.24	5.37	-1.2	0.71
TPn	*qTPn-A01*	A01	bin48	bin49	43.5	51.5	9,757,023	10,168,632	2.56	5	-0.62	1.4
TPn	*qTPn-A06*	A06	bin40	bin41	43.5	44.5	8,721,887	9,167,458	3.33	6.91	-0.12	-1.88
TPn	*qTPn-D12*	D12	bin187	bin188	148.5	149.5	55,359,867	55,595,846	3.33	7.03	0.07	1.92
SCi	*qSCi-A01*	A01	bin43	bin44	42.5	45.5	8,036,084	8,969,646	2.63	4.43	6.78	18.33
SCi	*qSCi-A04*	A04	bin182	bin183	145.5	146.5	57,244,810	57,548,850	2.78	5.03	1.72	-22.14
SCi	*qSCi-D04-1*	D04	bin32	bin33	28.5	32.5	4,450,078	4,794,145	3.19	5.67	-7.03	21.69
SCi	*qSCi-D04-2*	D04	bin131	bin132	86.5	88.5	46,227,657	47,544,347	2.94	4.94	-4.56	-20.82
SCi	*qSCi-D13*	D13	bin208	bin209	135.5	139.5	61,187,337	61,554,710	2.5	4.3	-14.5	1.89
TCi	*qTCi-A04*	A04	bin103	bin104	101.5	103.5	33,378,346	33,609,608	3.23	3.33	-3.67	-23.45
TCi	*qTCi-A11*	A11	bin7	bin55	14.5	53.5	807,428	9,258,460	2.75	12.52	-21.49	36.36
TCi	*qTCi-D04-1*	D04	bin32	bin33	28.5	32.5	4,450,078	4,794,145	2.86	2.95	-2.41	22.13
TCi	*qTCi-D04-2*	D04	bin131	bin132	86.5	88.5	46,227,657	47,544,347	3.32	3.21	-5.82	-21.48
SCond	*qSCond-A10*	A10	bin352	bin353	196.5	202.5	109,106,699	109,336,022	2.5	3.91	0.02	0.02
SCond	*qSCond-A12*	A12	bin423	bin425	158.5	159.5	98,555,400	99,115,052	3.91	6.42	0.03	-0.01
SCond	*qSCond-D04*	D04	bin142	bin143	88.5	94.5	50,189,434	50,421,587	2.59	4.03	-0.01	-0.03
SCond	*qSCond-D05*	D05	bin51	bin52	39.5	40.5	7,536,477	7,742,595	5.19	8.67	0.03	-0.01
SCond	*qSCond-D06*	D06	bin6	bin7	0	1.5	617,114	818,140	2.9	4.74	0.01	-0.04
SCond	*qSCond-D13*	D13	bin196	bin197	121.5	123.5	59,385,048	59,708,159	3.57	5.9	-0.03	0.01
TCond	*qTCond-A12*	A12	bin427	bin430	157.5	164.5	99,337,542	100,288,635	2.53	4.62	0.03	-0.02
TCond	*qTCond-D04-1*	D04	bin128	bin129	81.5	85.5	45,564,063	45,808,186	3.42	6.18	-0.01	-0.04
TCond	*qTCond-D04-2*	D04	bin131	bin132	86.5	87.5	46,227,657	47,544,347	3.62	6.52	-0.02	-0.04
STr	*qSTr-A05*	A05	bin119	bin120	95.5	103.5	20,625,360	21,148,783	2.82	3.3	0.58	1.03
STr	*qSTr-A07*	A07	bin285	bin286	141.5	145.5	88,546,004	88,983,661	2.84	3.3	0.76	-0.67
STr	*qSTr-A10*	A10	bin228	bin229	120.5	124.5	77,749,540	78,563,787	2.8	3.29	0.95	0.07
STr	*qSTr-A12*	A12	bin7	bin8	8.5	19.5	1,418,415	1,932,428	2.53	2.96	0.51	-0.89
STr	*qSTr-D02*	D02	bin66	bin63	55.5	59.5	11,571,331	11,240,351	3.8	4.38	-0.55	-1.17
STr	*qSTr-D04*	D04	bin23	bin24	16.5	18.5	2,781,571	3,052,412	2.77	3.34	-0.49	1.02
STr	*qSTr-D05*	D05	bin51	bin52	39.5	40.5	7,536,477	7,742,595	4.89	6.12	1.23	-0.11
STr	*qSTr-D06*	D06	bin6	bin7	0	1.5	617,114	818,140	3.74	4.63	0.15	-1.49
STr	*qSTr-D10*	D10	bin7	bin8	9.5	15.5	1,651,964	1,902,901	2.86	3.32	-0.91	-0.05
STr	*qSTr-D13*	D13	bin196	bin197	121.5	123.5	59,385,048	59,708,159	2.79	3.45	-0.87	0.11
TTr	*qTTr-A12*	A12	bin8	bin9	12.5	21.5	1,656,702	2,140,812	3.56	6.6	0.69	-1.07
Chl a	*qChl a-A13-1*	A13	bin270	bin278	145.5	159.5	85,560,449	86,656,939	2.73	4.64	-0.04	-0.03
Chl a	*qChl a-A13-2*	A13	bin286	bin287	173.5	184.5	80,629,132	83,806,916	2.69	5.21	-0.04	-0.01
Chl a	*qChl a-D01*	D01	bin239	bin240	136.5	146.5	62,926,804	63,199,901	3.05	5.09	-0.02	0.05
Chl a	*qChl a-D03*	D03	bin153	bin154	114.5	119.5	48,191,318	48,417,933	4.2	7.97	0	0.07
Chl a	*qChl a-D09*	D09	bin213	bin214	158.5	160.5	47,673,829	48,167,059	2.53	4.43	-0.04	0
Chl b	*qChl b-A13*	A13	bin289	bin290	173.5	184.5	87,115,053	87,473,165	2.7	5.01	-0.01	-0.01
Chl b	*qChl b-D01*	D01	bin239	bin240	136.5	146.5	62,926,804	63,199,901	2.79	5.22	-0.01	0.02
Chl b	*qChl b-D02*	D02	bin252	bin253	145.5	153.5	62,524,672	62,831,343	2.53	4.78	-0.01	0.02
Chl b	*qChl b-D03*	D03	bin153	bin154	114.5	119.5	48,191,318	48,417,933	3.97	8.1	-0.01	0.02
Car	*qCar-A12*	A12	bin314	bin315	89.5	93.5	76,853,357	77,247,271	2.84	5.57	0	0.01
Car	*qCar-D03*	D03	bin153	bin154	114.5	119.5	48,191,318	48,417,933	2.92	6.46	0	0.01
Chl a/b	*qChl a/b-D05*	D05	bin276	bin277	184.5	194.5	57,297,699	57,671,573	2.9	6.81	-0.05	-0.05
Chl a/b	*qChl a/b-D06*	D06	bin228	bin229	123.5	125.5	59,098,820	59,707,362	2.82	6.74	-0.05	0.04
Chl a/b	*qChl a/b-D12*	D12	bin172	bin173	125.5	138.5	52,342,567	53,232,613	2.56	6.27	-0.05	0.05
Total Chl	*qTotal Chl-A13*	A13	bin48	bin49	47.5	49.5	5,904,987	6,263,087	2.51	4.46	-0.05	-0.01
Total Chl	*qTotal Chl-D01*	D01	bin238	bin239	136.5	147.5	62,826,804	63,033,940	2.67	4.49	-0.03	0.06
Total Chl	*qTotal Chl-D03*	D03	bin153	bin154	114.5	118.5	48,191,318	48,417,933	4.99	9.49	-0.02	0.1
PH2	*qPH2-A11*	A11	bin414	bin439	258.5	264.5	92,237,505	97,642,814	2.66	4.32	0.32	-15.33
PH2	*qPH2-D01*	D01	bin209	bin210	111.5	117.5	59,207,021	59,470,354	2.73	4.04	7.05	10.07
PH2	*qPH2-D03*	D03	bin153	bin154	114.5	119.5	48,191,318	48,417,933	4.06	6.47	-13.14	-0.61
PH2	*qPH2-D06*	D06	bin59	bin60	60.5	66.5	7,355,757	7,642,365	2.66	3.94	9.93	4.05
PH2	*qPH2-D09*	D09	bin215	bin216	158.5	165.5	48,167,059	48,412,567	2.54	3.77	4.15	11.91
PH2	*qPH2-D12*	D12	bin221	bin222	189.5	192.5	60,587,231	60,897,033	4.86	7.87	14.21	-1.36
FBN	*qFBN-A04*	A04	bin39	bin40	58.5	71.5	7,802,365	8,134,366	2.64	3.82	1.13	-0.96
FBN	*qFBN-A05*	A05	bin44	bin45	34.5	35.5	7,019,176	7,291,976	3.58	5.29	0.47	-1.89
FBN	*qFBN-A06*	A06	bin22	bin23	26.5	35.5	3,658,624	4,677,844	3.97	6.64	0.23	-2.18
FBN	*qFBN-D04*	D04	bin51	bin52	45.5	48.5	7,626,894	7,826,894	2.8	3.88	0.72	-1.34
FBN	*qFBN-D11*	D11	bin89	bin90	73.5	75.5	14,642,790	15,006,002	3.44	5.2	-0.37	-1.83
FBN	*qFBN-D12*	D12	bin106	bin107	77.5	78.5	41,087,553	41,476,816	3.4	5.05	-1.33	0.37
BN	*qBN-D05*	D05	bin104	bin105	70.5	76.5	15,695,738	16,137,583	2.55	4.66	-2.13	-2.04
BN	*qBN-D10*	D10	bin11	bin22	19.5	43.5	2,290,240	4,417,087	2.85	5.34	2.58	1.96
SCY	*qSCY-D07*	D07	bin231	bin232	188.5	189	57,783,341	58,417,178	2.53	5.54	1.92	12.2
SCY	*qSCY-D10*	D10	bin22	bin21	21.5	45.5	4,200,666	4,417,087	2.67	5.85	8.64	7.69
LY	*qLY-D07*	D07	bin231	bin232	188.5	189	57,783,341	58,417,178	2.64	6.04	0.74	3.6
BW	*qBW-A10*	A10	bin57	bin58	48.5	52.5	10,608,267	10,844,347	3.55	7.6	-0.32	-0.22
SI	*qSI-D01*	D01	bin101	bin103	73.5	74.5	19,943,730	21,931,208	2.68	6.14	0.05	0.91
SI	*qSI-D05*	D05	bin10	bin11	8.5	10.5	2,123,451	2,323,451	3.7	8.56	0.8	0.13
LP	*qLP-A07*	A07	bin107	bin108	83.5	87.5	19,417,639	19,813,491	2.66	4.84	-0.56	-2.13
LP	*qLP-D09*	D09	bin221	bin222	164.5	165.5	49,209,126	49,409,127	3.48	6.77	-1.94	-0.37
LP	*qLP-D10*	D10	bin22	bin21	36.5	45.5	4,200,666	4,417,087	4.43	8.69	-2.48	0.53
LI	*qLI-D09*	D09	bin225	bin226	168.5	169.5	49,640,725	49,892,967	3.41	6.69	-0.44	-0.05
LI	*qLI-D10*	D10	bin21	bin54	36.5	52.5	4,200,666	10,690,859	3.76	8.08	-0.54	0.14
LI	*qLI-D12*	D12	bin191	bin192	149.5	155.5	55,816,594	56,150,659	2.83	5.17	0.32	-0.3
FL	*qFL-A06*	A06	bin26	bin27	35.5	39.5	5,979,701	6,426,275	2.92	13.58	-0.94	0.85
FL	*qFL-A10*	A10	bin53	bin50	49.5	51.5	9,611,144	9,322,125	2.82	14.17	0.99	-0.94
FL	*qFL-D12*	D12	bin109	bin110	75.5	84.5	41,741,946	42,083,775	2.53	11.9	1.17	-0.25
FS	*qFS-A02*	A02	bin279	bin280	152.5	156.5	104,826,173	105,601,237	5.37	17.11	0.21	2.46
FS	*qFS-A11*	A11	bin129	bin130	108.5	110.5	21,948,343	22,148,343	2.96	8.55	0.28	-1.65
FS	*qFS-D02*	D02	bin94	bin95	68.5	75.5	19,742,795	20,003,289	3.74	11.13	0.3	-1.95
FS	*qFS-D12*	D12	bin154	bin155	124.5	127.5	49,828,942	50,092,227	7.51	24.89	2.34	-0.74
MIC	*qMIC-A13*	A13	bin343	bin344	206.5	208.5	96,488,822	97,053,406	2.93	14.4	0.31	0.05
MIC	*qMIC-D01*	D01	bin58	bin59	51.5	54.5	10,890,905	11,226,434	4.44	21.98	-0.37	0.2
MIC	*qMIC-D02*	D02	bin3	bin4	5.5	7.5	620,059	1,091,799	2.63	11.75	0.03	-0.39
MIC	*qMIC-D03*	D03	bin10	bin11	15.5	18.5	1,178,422	1,526,855	3.05	12.35	0.22	-0.3
FU	*qFU-A13*	A13	bin233	bin234	134.5	136.5	69,500,824	70,270,368	6.86	20.51	0.26	-1.6
FU	*qFU-D03*	D03	bin13	bin14	22.5	29.5	1,728,364	2,715,388	3.17	8.54	0.68	0.32
FU	*qFU-D06*	D06	bin37	bin38	40.5	47.5	4,733,957	4,933,957	3.82	10.62	0.08	-1.24
FU	*qFU-D12-1*	D12	bin10	bin11	16.5	22.5	59,195,554	59,480,927	4.54	12.67	0.22	-1.3
FU	*qFU-D12-2*	D12	bin212	bin213	170.5	175.5	1,425,534	1,957,383	5.15	15.29	0.12	1.38
FE	*qFE-A10*	A10	bin217	bin218	115.5	117.5	75,292,930	75,555,428	2.55	7.69	0.19	-0.19
FE	*qFE-D05-1*	D05	bin94	bin95	69.5	74.5	60,252,767	60,452,767	4.08	11.71	-0.26	-0.37
FE	*qFE-D05-2*	D05	bin295	bin296	205.5	207.5	14,282,823	14,559,501	2.6	7.86	-0.2	-0.13
FE	*qFE-D09*	D09	bin131	bin151	86.5	99.5	33,095,130	37,366,030	3.76	20.3	0.39	-0.44

**Table 4:** Sixteen QTLs detected in this study overlapped with that identified in previous studies

This Study				Previous Report							
**Name**	**Chr**	**Start**	**End**	**Reported**	**Start**	**End**	**QTL**	**LOD**	**R**^**2**^	**Parents**	**Ref**
FBN	D04	7,626,894	7,826,894	FBN	4,447,115	8,199,618	*qFBN-22–2*	3.84	17.3	TM-1*CSB22sh	[46]
BN	D05	15,695,738	16,137,583	BN	15,468,703	17,887,617	*qBN-D5-1*	1.66	3.5	(STV2B*Foster6)*(DPL15*CRI7)	[47]
LP	D10	4,200,666	4,417,087	LP	4,294,769	5,493,007	*Cs9_PF_20_(5.27* +*)、Cs8_PF_20_(6.57* +*)*	5.27	19.10	Guazuncho2(Gh)*VH8-4602(Gb)	[48]
FL	A06	5,979,701	6,426,275	FL	4,440,391	6,580,618	*qFLchr6*	4.54	7.80	(Emian22*3–79)*Emian22	[49]
FL	A06	5,979,701	6,426,275	FL	4,439,492	6,580,618	*qChr06FL*	NA	NA	(Emian22*3–79)*Emian22	[50]
FL	A10	9,611,144	9,322,125	FL	7,964,148	10,069,511	qFL-A10-1*	3.33	6.33	Zhongmiansuo12*8891	[51]
FL	A10	9,611,144	9,322,125	FL	7,964,148	10,069,511	*qFL-A10-1_*	4.32	10.46	Zhongmiansuo12*8891	[52]
FS	A02	104,826,173	105,601,237	FS	104,227,599	105,227,815	*qFS-C2-1*	3.95	5.69	Lumianyan22*Luyuan343	[53]
FS	D02	19,742,795	20,003,289	FS	19,710,781	20,711,127	*qFS-D2-1*	8.12	14.4	(Simian3*Sumian12)*(Zhong4133*8891)	[54]
FM	D02	620,059	1,091,799	FM	456,594	1,457,031	*qFM-D2-1*	3.59	6.80	(Simian3*Sumian12)*(Zhong4133*8891)	[54]
FM	D02	620,059	1,091,799	FM	991,691	3,224,078	*qFM-D2-2*	12.82	21.92	CRI12*J8891	[55]
FM	D02	620,059	1,091,799	FM	456,594	3,223,772	*qFMIC-D2-1_*	3.32	9.1	Zhongmiansuo12*8891	[52]
FM	D02	620,059	1,091,799	FM	456,594	3,224,164	qFMIC-D2-1*	8.01	12.49	Zhongmiansuo12*8891	[51]
FU	D03	1,728,364	2,715,388	FU	2,695,254	3,695,267	*TC-qFU-c17-2*	3.11	5.7	CRI36*Hai7124	[56]
FE	A10	75,292,930	75,555,428	FE	72,032,342	79,020,626	*qFE-10-1b*	30.93	11.8	HS427-10*TM-1	[57]
FE	D05	14,282,823	14,559,501	FE	13,336,541	14,337,210	*qFE-D5-1*	2.91	7.49	Zhongmiansuo12*8891	[52]

Twelve QTLs were associated with plant type traits at seedling stage, most of which (75%) had positive effects and originated from TM-1, suggesting that *G. hirsutum* has a growth advantage in the seedling stage. Among these QTLs, the PVE varied from 4.46 to 8.35%; the QTL *qCNH-A12* with the highest PVE (8.35%) had positive effects and came from Hai7124. Thirty-seven QTLs were detected for leaf morphology and physiological traits at seedling stage, featuring positive effects and coming from both TM-1 and Hai7127 (19 and 18 QTLs, respectively). We found that all nine QTLs associated with intracellular CO_2_ concentration had positive effects and originated with TM-1, and 7/9 demonstrated positive effects, which is the main component of heterosis. A total of 17 QTLs were identified for leaf chlorophyll, with PVE values ranging from 4.43 to 8.1%; both the additive and dominant effects of these QTLs were close to 0.

**Fig. 3:**
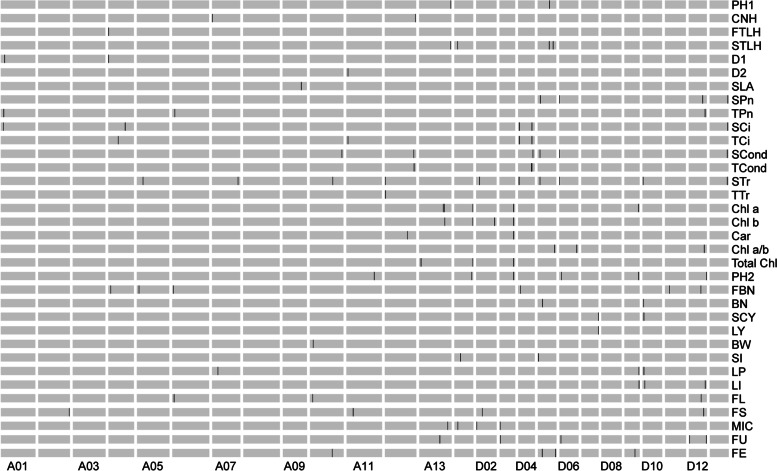
Chromosomal distribution of QTLs associated with 35 traits. Black lines indicate QTL positions on the chromosomes

Twenty-six QTLs were identified for yield or yield-related traits. Most QTLs associated with qPH2 and qFBN, and all those with qSCY, qLY, and qSI, exhibited positive effects and came from Hai7124. Meanwhile, QTLs having positive effects associated with qBW, qLP, and qLI came from TM-1, suggesting that *G. barbadense* has a larger biomass but *G. hirsutum* has higher fiber yield. Of QTLs associated with fiber quality traits, 80% of those having positive effects came from Hai7124; only four QTLs (*qFL-A06*, *qMIC-D01*, *qFE-D05-1*, and *qFE-D05-2*) with positive effects originated from TM-1. This result indicated that the genetics governing excellent fiber quality come from *G. barbadense*. All QTLs and the corresponding location information, LOD, PVE, additive effect, and dominant effect values were presented in Table 3 and Fig. 3.

### Candidate gene identification and expression analysis

We identified ten genes that has nonsynonymous SNPs in exons or SNP in their upstream regions was located within the 15 loci of interest for 14 traits (D1, CHN, FLTH, PH1, Sci, TCi, Tcond, BW, SCY, LP, Li, SPn, TPn, TTr). We analyzed their expression in sixteen vegetative and reproductive tissues of TM-1 and compared values with those in Hai7124 (Supplementary Fig. 2, Supplementary Table 4, 5). Some SNP variants corresponding to QTLs associated with different traits were mapped to the same position or related to the same gene, such as *GH_A04G0054*/*GB_A04G0055*, *GH_D04G1426*/*GB_D04G1512*, and *GH_D10G0500*/*GB_D06G1730* (Supplementary Table 4, 5).

**Fig. 4:**
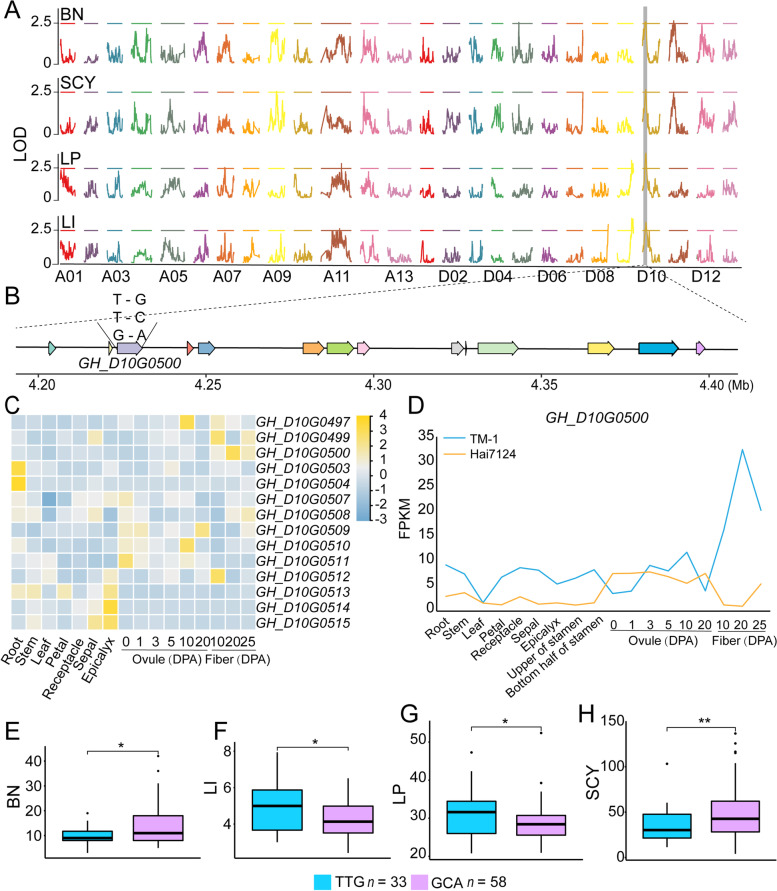
Functional haplotypes in associated loci from the TM-1 × Hai7124 F2 population on D10. **A** Genetic mapping of a QTL on the D10 chromosome identified as related to BN, SCY, LP, and LI. **B** Genes with nonsynonymous SNPs in the QTL region. **C** Transcriptomic expression of QTL-region genes with nonsynonymous SNPs in TM-1 tissues, based on FPKM values. **D** Transcriptomic expression of GH_D10G0500 in TM-1 and Hai7124 tissues, based on FPKM values. **E–H** Boxplot of GH_D10G0500 haplotypes. Center line, median; box limits, upper and lower quartiles; whiskers, 1.5 × the interquartile range; dots, outliers (* P < 0.01, ** P < 0.001, two-tailed t-test)

A representative QTL that related to multiple traits BN, SCY, LP, and LI was located on chromosome D10 (Fig. 4A). This locus encompassed fourteen genes harboring nonsynonymous SNPs. Considering the expression of these genes during fiber development, one was identified as a putative causal gene: root hair defective 3 GTP-binding protein (*GhRHD3*, *GH_D10G0500*), which was dominantly expressed during secondary cell-wall bio-synthesis (20 DPA) (Fig. 4B-C). Interestingly, its Hai7124 homolog showed high expression during fiber initiation (0, 1, and 3 DPA) (Fig. 4D). Three nonsynonymous SNPs in *GH_D10G0500*, D10Gh: 4,228,677/4228733/4229273 (TTG versus GCA 33:58), demonstrated significant associations with BN, SCY, LP, and LI (Fig. 4E-H). The orthologous gene in *Arabidopsis* thaliana was identified as involved in the regulation of cell expansion [58–60]

Supplementary Fig. 3 illustrates a QTL, located on chromosome D04, which related to SCi, TCi, and Tcond. There were thirteen genes harboring nonsynonymous SNPs in this region. Combining their expression level during fiber development, one was identified as a putative causal gene: glycerol-3-phosphate acyltransferase 6 (*GhGPAT6*, *GH_D04G1426*), which was highly expressed in leaves. Within this gene, the nonsynonymous SNP D04Gh:47,064,565 (CC versus AA 65:50) was significant associated with SCi, TCi, and Tcond. As reported, its orthologous gene in tomato was involved in regulating the outer wall diameter of leaf epidermal cells [61].

## Discussion

### Bin markers are effective for constructing high-density genetic maps and QTL fine mapping in *G. hirsutum* and *G. barbadense*

In recent years, scientists have used specific-locus amplified fragment sequencing (SLAF-seq), genotyping by sequencing (GBS), and other sequencing methods to genotype the complex genome of cotton, and the resulting genetic map is based on SNP phasing. This method can identify markers with high throughput; in addition, the chromosome coverage is more uniform and the marker density greatly improved compared with traditional PCR-based markers. With the help of newly developed bioinformatics software, it is possible to complete genotyping and construct genetic maps in a very short time. Compared to the GBS-based enzyme digestion method, a binmap based on resequencing offers the following improvements: scanning of and mutation identification at all sites in the whole genome, without any prior marker information, yielding complete allelic variant information with higher accuracy than previous experimental methods. In this study, we obtained a total of 6303 high-confidence bin markers that not only extends the length of the cotton genetic map but also improve its resolution. In our previous studies, we constructed an SSR-based genetic map using a (TM-1 × Hai7124)F_2_ population [27] that spanned 3414 loci in 26 linkage groups, covering 3667.62 cM with an average inter-locus distance of 1.08 cM. The present 6303 bin markers expand that map to cover 5057.13 cM while also narrowing the average distance between adjacent markers on to an interval of 0.8 cM. The bin marker length varies from 0.64 to 1.10 Mb, which indicates that the final location of a QTL can be reduced to dozens of candidate genes. Thanks to the binmap algorithm, SNPs within a haplotype can be corrected, decreasing the false positive possibility of a single SNP. The binmap also allows obtaining the fragments of the tested samples on the whole chromosome to exchange recombination information.

### Most traits show heterosis in F_1_ and F_2_

In most F_2_ populations, the traits exhibited by individual plants fall between those of their parents, while a few exceed their parents; thus, most traits demonstrate different degrees of over-parental segregation. Such phenotypic trait data exhibits an approximately normal distribution. Each pair of the 35 traits was evaluated for significant negative or positive correlations (Supplementary Fig. 2), and we also evaluated the heterosis of each trait in the F_1_ and F_2_ populations. In the F_1_ population, BN exhibited the highest mid-parent heterosis of all yield traits, at 59.7%; other yield traits ranked LY > SCY > LI > SI > LP, while the mid-parent heterosis of BW was negative. Accordingly, the mid-parent heterosis of BN contributes most to the heterosis of yield. With respect to plant type, morphology, and physiological traits, the mid-parent heterosis of SLA was the highest at 40.49%, followed by TLA at 36.16%, and then other traits in the range of 2.1%-23.81 except for D1, which had a negative value. Regarding fiber quality traits, these ranked in terms of mid-parent heterosis as FS > FL > FE, with the values for MIC and FU being negative (Supplementary Table 3). In the F_2_ population, both yield and quality traits exhibited negative mid-parent heterosis values. Among plant type, morphology, and physiological traits, most mid-parent heterosis values were positive, ranging from 1.18% to 23.81% except for the values associated with PH1, STLH, D2, TLA, Car, and Chl a/b. (Supplementary Table 3; Table 2).

### Non-uniform distribution of QTLs in the A and D subgenomes

In this study, a total of 112 QTLs were detected, of which 71 were in the D subgenome, much more than the 41 in the A subgenome (Table S6). Of QTLs associated with the six plant type traits at seedling stage, more were sited in the A subgenome than in the D subgenome; in contrast, QTLs associated with the other ten leaf morphology and physiological traits at seedling stage, five traits reflecting leaf chlorophyll content, two plant type traits at flower and boll stage, seven yield traits, and five fiber quality traits were all less commonly located in the A subgenome than in the D subgenome. In particular, the D subgenome showed a strong advantage with regard to leaf chlorophyll content, yield traits, and fiber quality traits. This is consistent with previous reports that the D subgenome contributes more to the genetic control of fiber [62–64], and suggests that molecular marker selection in the D subgenome may be more efficient for breeding to improve yield and fiber quality.

### QTLs and candidate genes may contribute to the improvement of cotton through breeding

Studies involving cotton QTL mapping and candidate gene identification generally focus on traits related to yield and fiber quality; considerably less research has been conducted concerning seedling traits, leaf physiology, and chlorophyll content. Nonetheless, these traits are also important for cotton growth: plant height and leaf area at the seedling stage determine growth vigor, which in turn affects adversity resistance; meanwhile, leaf physiological and chlorophyll content can enhance photosynthesis efficiency and solar energy utilization, eventually helping adaptation to dense planting and increasing production. Here, the candidate gene *GH_D04G1426* demonstrated significant associations with SCi, TCi, and Tcond. Its orthologous gene in tomato has been reported to affect the outer wall diameter of leaf epidermal cells; such functionality may indirectly affect photosynthesis in cotton [61]. In looking beyond direct effects on yield and fiber quality, other QTL and candidate genes in our data may provide additional solutions for cotton molecular breeding.

## Conclusions

In conclusion, we constructed a high-density genetic map based on the resequencing data of 249 individuals from an interspecific F_2_ population (TM-1 and Hai7124). This genetic map consists of 6303 high-confidence bin markers spanning 5057.13 cM across 26 chromosomes. Based on this map, 112 QTLs relating to agronomic and physiological traits from seedling to boll opening stage were identified. Through the analysis of sequence and expression of the candidate genes within the QTLs mapping regions, ten causal putative genes might responsible for the target traits. Of them, *GhRHD3* (*GH_D10G0500*) was associated with fiber yield and *GhGPAT6* (*GH_D04G1426*) might play important role in photosynthesis efficiency.

## Supplementary Information

**Additional file 1:** **Supplementary Figure 1. **Comparisons of the TM-1 genome with (TM-1 × Hai7124) F2 genetic map. **Supplementary Figure 2. **Frequency distribution of phenotypic variation of 30 traits and correlation coefficients among the traits in the F2 population. **Supplementary Figure 3. **Functional haplotypes in associated loci from the TM-1×Hai7124 F2 population on D10. **Supplementary Figure 4. **Functional haplotypes in associated loci from the TM-1×Hai7124 F2 population on D04. **Supplementary Table 1. **The distribution characteristics of partial segregation markers. **Supplementary Table 2. **Shortened form and Unit of measurement of the 35 traits. **Supplementary Table 3.** Statistical analysis of 35 traits phenotypic differences in TM-1 Hai7124 and F1. **Supplementary Table 4. **Haplotype and gene ID of the 10 candidate genes in TM-1 and Hai7124. **Supplementary Table 5. **Expression of the 10 candidate genes in different tissues of TM-1 and Hai7124. **Supplementary Table 6. **The distribution of QTLs in the At and Dt subgenomes.

**Additional file 2: Table S7.** All markers in geneticmap.

## Data Availability

The raw sequencing data used in this study are available from the China National GenBank (CNGB) Nucleotide Sequence Archive (CNSA) under accession number sub026937.

## Abbreviations

RAD-seq: Restriction-site Associated DNA sequence
SNPs: Single Nucleotide Polymorphism
QTL: Quantitative Trait Locus
CTAB: Cetyltriethylammnonium Bromide
SSR-PCR: Simple Sequence Repeats PCR
PH1: Plant Height
CNH: Cotyledonary Node Height
FTLH: First True Leaf Height
STLH: Second True Leaf Height
D1: Distance between the cotyledonary node and first true leaf
D2: Distance between first true leaf and second true leaf
Chl a: Chlorophyll a
Chl b: Chlorophyll b
Car: Carotenoid
Chl a/b: Chlorophyll a/b ratio
Total Chl: Total Chlorophyll
PH2: Plant Height
FBN: Fruit Branch Number
BN: Boll Number per plant
SCY: Seed Cotton Yield
LY: Lint Yield
BW: Boll Weight
LP: Lint Percentage
LI: Lint Index
SI: Seed Index
FL: Fiber Length
FS: Fiber Strength
MIC: Micronaire Value
FU: Fiber length Uniformity
FE: Fiber Elongation
GATK4: Genome Analysis Toolkit 4
MAF: Minor Allele Frequency
H: Heterosis
A: The additive effect
D: Dominant effect
R2: Contribution rate
