# Supplementary material for: Construction of a high-density genetic map and identification of QTLs related to agronomic and physiological traits in an interspecific (Gossypium hirsutum × Gossypium barbadense) F2 population
Source: BMC Genomics. 2022 Apr 15;23:307. doi: 10.1186/s12864-022-08528-2 (PMC9013169; doi:10.1186/s12864-022-08528-2)
Supplement: Supplementary file 1 — Additional file 1: Supplementary Figure 1. Comparisons of the TM-1 genome with (TM-1 × Hai7124) F2 genetic map. Supplementary Figure 2. Frequency distribution of phenotypic variation of 30 traits and correlation coefficients among the traits in the F2 population. Supplementary Figure 3. Functional haplotypes in associated loci from the TM-1×Hai7124 F2 population on D10. Supplementary Figure 4. Functional haplotypes in associated loci from the TM-1×Hai7124 F2 population on D04. Supplementary Table 1. The distribution characteristics of partial segregation markers. Supplementary Table 2. Shortened form and Unit of measurement of the 35 traits. Supplementary Table 3. Statistical analysis of 35 traits phenotypic differences in TM-1 Hai7124 and F1. Supplementary Table 4. Haplotype and gene ID of the 10 candidate genes in TM-1 and Hai7124. Supplementary Table 5. Expression of the 10 candidate genes in different tissues of TM-1 and Hai7124. Supplementary Table 6. The distribution of QTLs in the At and Dt subgenomes. [file 12864_2022_8528_MOESM1_ESM.docx]

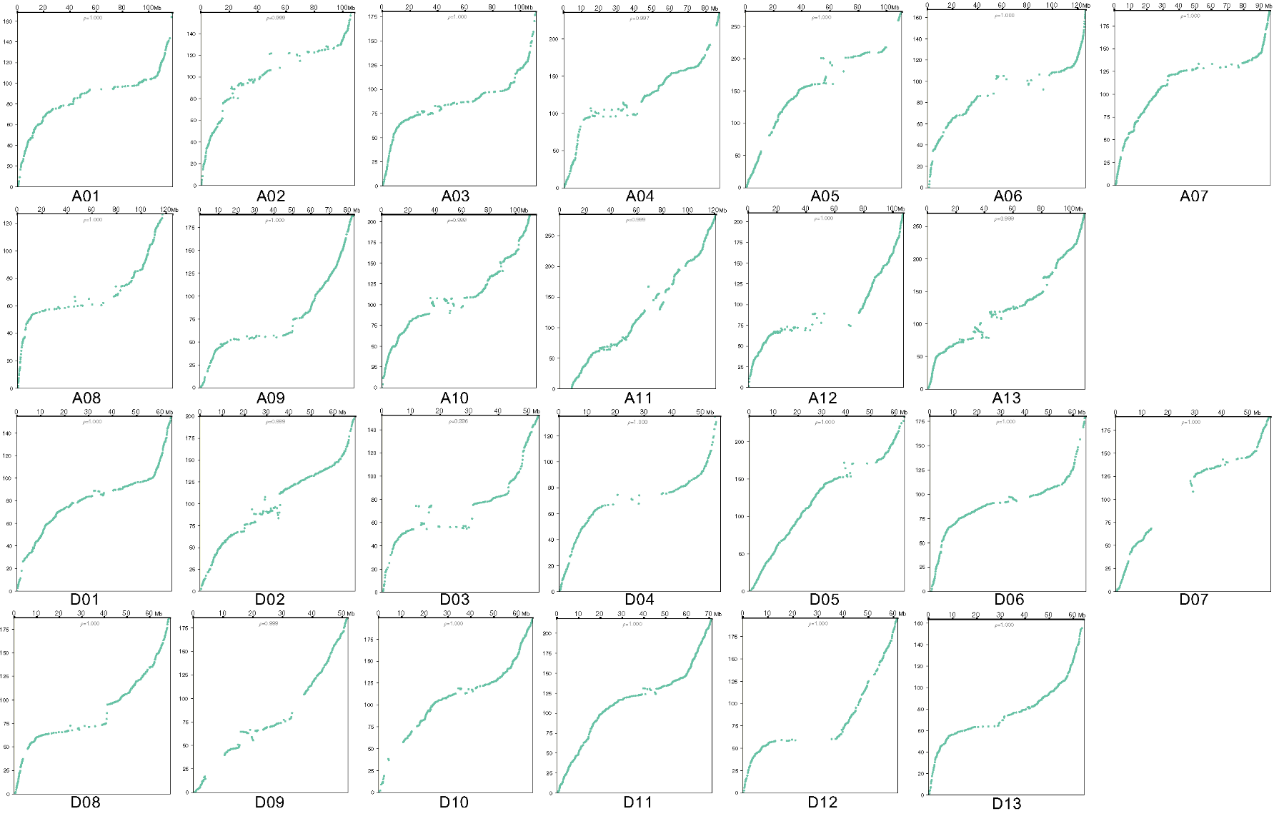


**Supplementary Figure 1 Comparisons of the TM-1 genome with (TM-1 × Hai7124) F_2_ genetic map.** Collinearity analysis of bin markers between the physical map and genetic map in TM-1 genome. The x and y axes represent the physical sequences (in megabases) and genetic distances (in centimorgans), respectively.


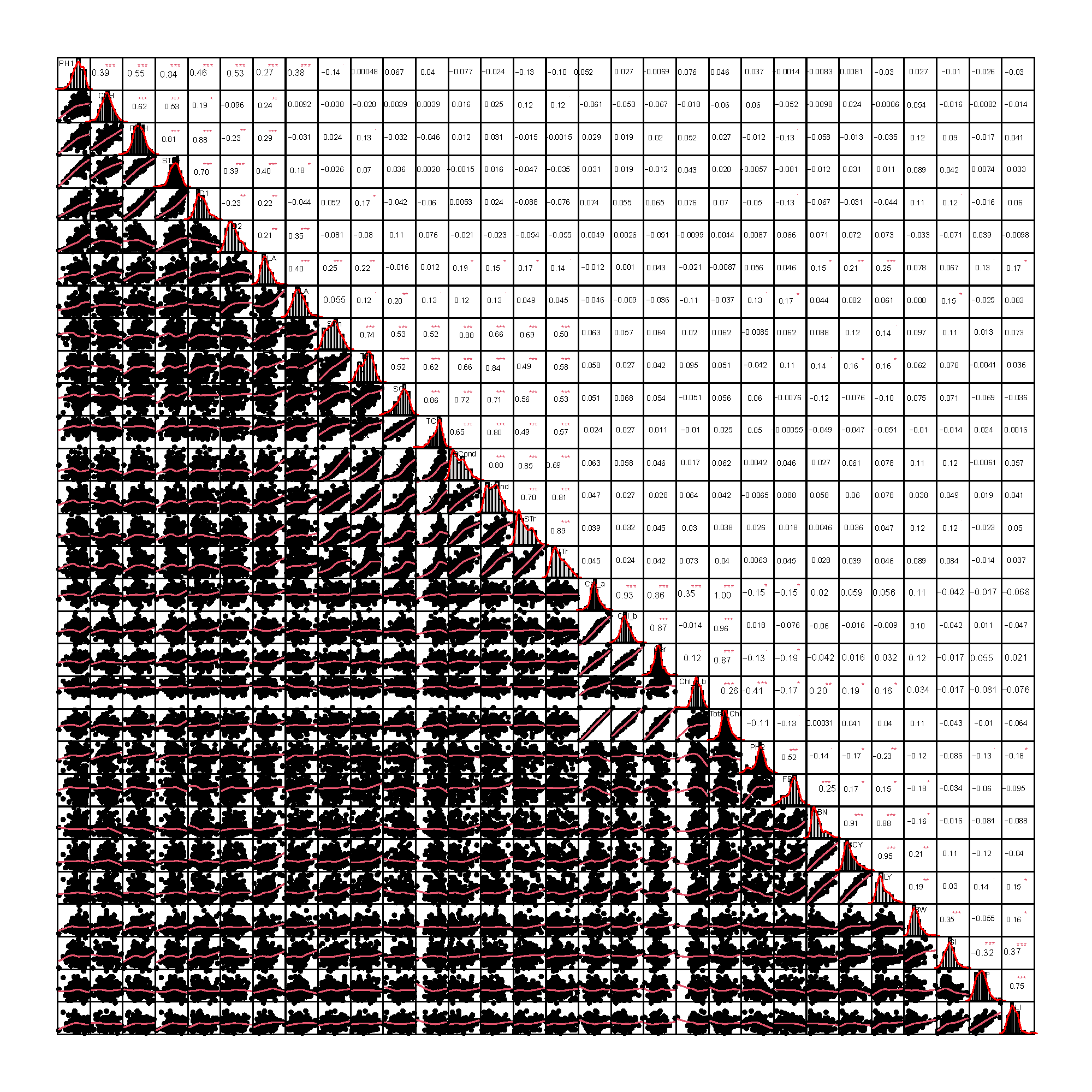
 **Supplementary Figure 2 Frequency distribution of phenotypic variation of 30 traits and correlation coefficients among the traits in the F_2_ population.**

*indicates significant difference at P = 0.05; **indicates extremely significant difference at P = 0.01; *** indicates extremely significant difference at P = 0.001.


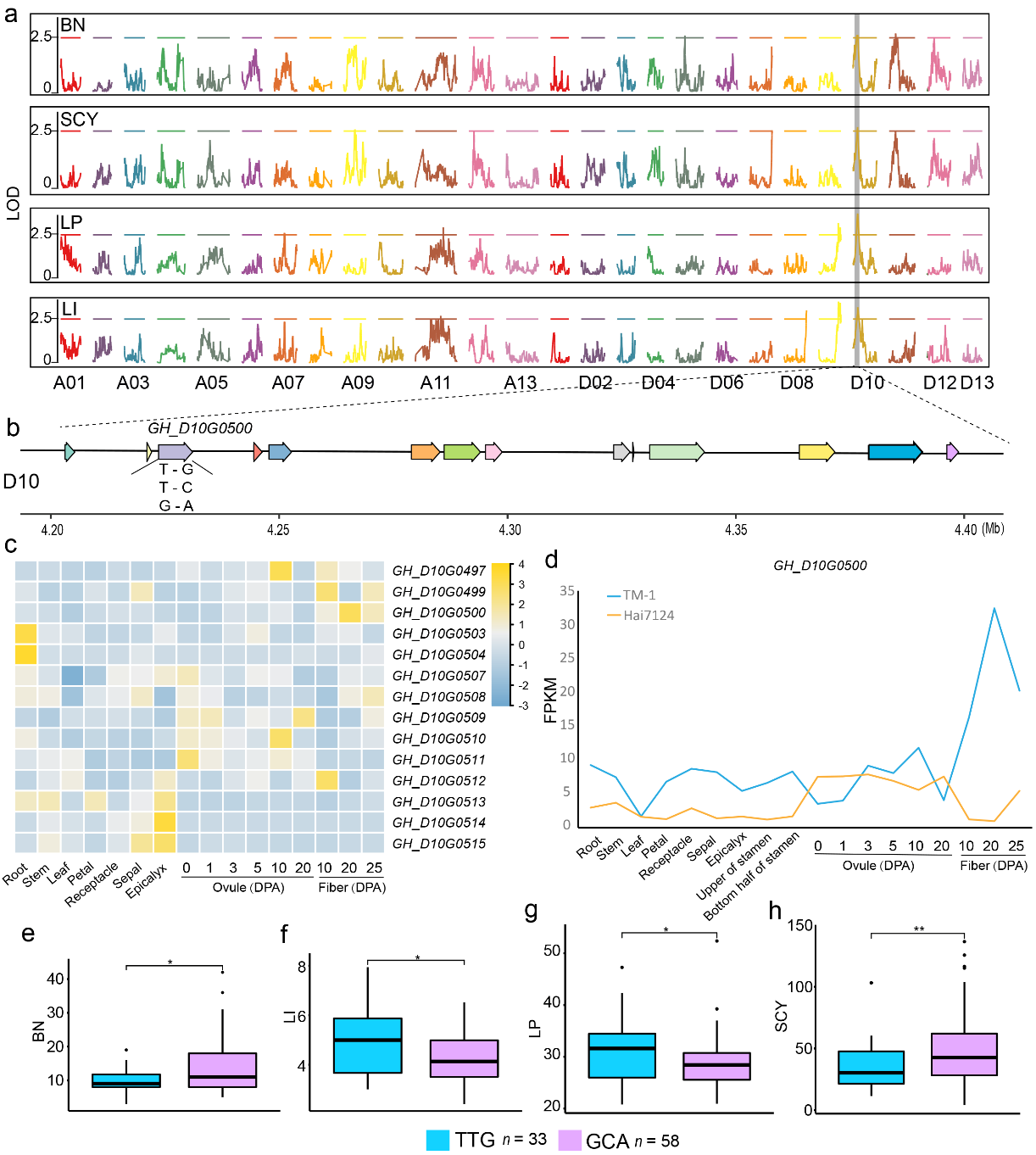


**Supplementary Figure 3. Functional haplotypes in associated loci from the TM-1×Hai7124 F_2_ population on D10.** (a) Genetic mapping of a QTL on the D10 chromosome identified as related to BN, SCY, LP, and LI. (b) Genes with nonsynonymous SNPs in the QTL region. (c) Transcriptomic expression of QTL-region genes with nonsynonymous SNPs in TM-1 tissues, based on FPKM values. (d) Transcriptomic expression of *GH_D10G0500* in TM-1 and Hai7124 tissues, based on FPKM values. (e-h) Boxplot of *GH_D10G0500* haplotypes. Center line, median; box limits, upper and lower quartiles; whiskers, 1.5x the interquartile range; dots, outliers (* *P*<0.01, ** *P*<0.001, two-tailed *t*-test).


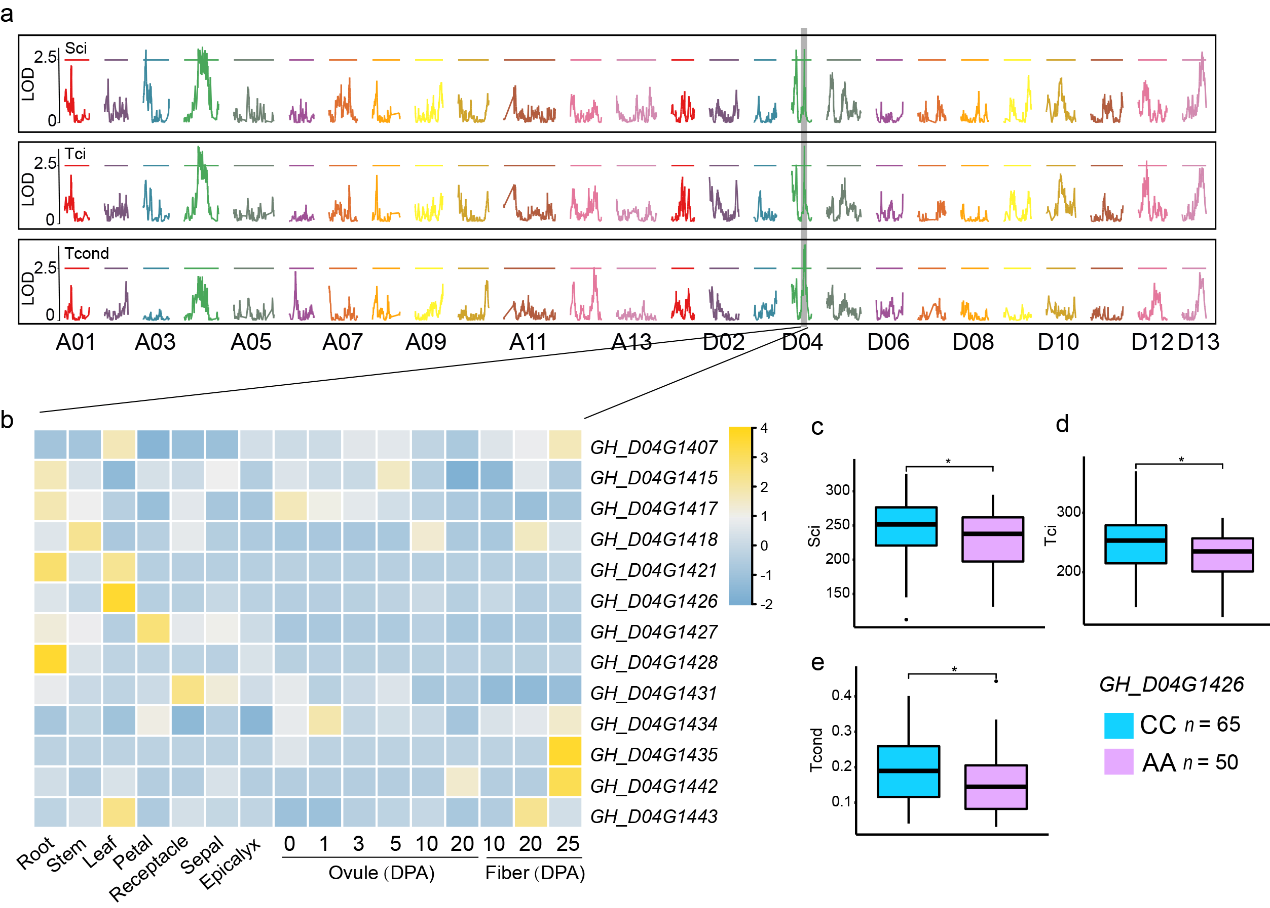


**Supplementary Figure 4 Functional haplotypes in associated loci from the TM-1×Hai7124 F2 population on D04.** (a) Genetic mapping of a QTL on the D04 chromosome identified as related to SCi, TCi, and TCond. (b) Transcriptomic expression of QTL-region genes with nonsynonymous SNPs in TM-1 tissues, based on FPKM values. (c-e) Boxplot of *GH_D04G1426* haplotypes. Centerline, median; box limits, upper and lower quartiles; whiskers, 1.5x the interquartile range; dots, outliers (* *P*<0.01, two-tailed *t*-test).

| Chromosome | Toward TM-1 | Toward Hai7124 | Toward F_1_ | Ratio (partial segregation bins/total bins in the chromosome) |
| --- | --- | --- | --- | --- |
| A01 | 2 | 0 | 0 | 0.85 |
| A02 | 4 | 21 | 0 | 12.69 |
| A03 | 0 | 1 | 0 | 0.39 |
| A04 | 1 | 34 | 0 | 14.64 |
| A05 | 0 | 89 | 0 | 34.77 |
| A06 | 14 | 8 | 0 | 10.68 |
| A07 | 5 | 10 | 0 | 5.88 |
| A08 | 4 | 35 | 0 | 20.63 |
| A09 | 1 | 9 | 0 | 3.82 |
| A10 | 11 | 30 | 0 | 12.58 |
| A11 | 10 | 116 | 0 | 31.11 |
| A12 | 21 | 2 | 0 | 8.10 |
| A13 | 7 | 15 | 0 | 6.32 |
| At-total | 80 | 370 | 0 | 13.02 |
| D01 | 0 | 1 | 0 | 0.43 |
| D02 | 2 | 2 | 0 | 1.56 |
| D03 | 1 | 1 | 0 | 1.16 |
| D04 | 1 | 17 | 0 | 11.46 |
| D05 | 1 | 5 | 0 | 2.13 |
| D06 | 0 | 19 | 0 | 8.60 |
| D07 | 0 | 55 | 0 | 31.79 |
| D08 | 0 | 23 | 0 | 10.6 |
| D09 | 0 | 55 | 0 | 29.73 |
| D10 | 1 | 50 | 0 | 22.37 |
| D11 | 0 | 10 | 0 | 3.15 |
| D12 | 0 | 23 | 0 | 11.79 |
| D13 | 0 | 7 | 0 | 3.32 |
| Dt-Total | 6 | 268 | 0 | 9.62 |
| Total | 86 | 638 | 0 | 11.49 |

**Supplementary Table 1 The distribution characteristics of** **partial segregation markers**

| Traits | Shortened form | Unit of measurement |
| --- | --- | --- |
| Plant height | PH1 | cm |
| Cotyledonary node height | CNH | cm |
| First true leaf height | FTLH | cm |
| Secend true leaf height | STLH | cm |
| Distance between cotyledonary node and first true leaf | D1 | cm |
| Distance between first true leaf and secend true leaf | D2 | cm |
| Secend true leaf area | SLA | cm2 |
| Third true leaf area | TLA | cm2 |
| Secend true leaf Photosynthesis ratio | SPn | μmolCO2·m-2·s-1 |
| Third true leaf Photosynthesis ratio | TPn | μmolCO2·m-2•s-1 |
| Secend true leaf Intercellular CO2 concentration | SCi | μmol·mol-1 |
| Third true leaf Intercellular CO2 concentration | TCi | μmol•mol-1 |
| Secend true leaf Stomatal conductance | SCond | mmol·m-2·s-1 |
| Third true leaf Stomatal conductance | TCond | mmol•m-2•s-1 |
| Secend true leaf Transpiration rate | STr | g•m-2•h-1 |
| Third true leaf Transpiration rate | TTr | g·m-2·h-1 |
| Chlorophyll a | Chl a | mg/g |
| Chlorophyll b | Chl b | mg/g |
| Carotenoid | Car | mg/g |
| Chlorophyll a/b | Chl a/b |  |
| Total chlorophyll | Total Chl |  |
| Plant height | PH2 | cm |
| Fruit branch number | FBN |  |
| Bolls/Plant | BN |  |
| Seed cotton yield | SCY | g |
| Lint yield | LY | g |
| Boll weight | BW | g |
| Seed index | SI | g |
| Lint percentage | LP | % |
| Lint index | LI | g |
| Fiber length | FL | mm |
| Fiber strength | FS | cN /dtex |
| Micronaire | MIC |  |
| Fiber length uniform | FU |  |
| Fiber elongation | FE |  |

**Supplementary Table 2 Shortened form and Unit of measurement of the 35 traits**

| Traits | TM-1 | | |  | H7124 | | |  | F_1_ | | | mid-parent heterosis |
| --- | --- | --- | --- | --- | --- | --- | --- | --- | --- | --- | --- | --- |
|  | Mean±SD | 5% | 1% |  | Mean±SD | 5% | 1% |  | Mean±SD | 5% | 1% |  |
| PH1 | 15.61±3.35 | a | A |  | 26.99±2.04 | b | B |  | 23.02±0.84 | b | B | 8.08 |
| CNH | 6.49±0.64 | a | A |  | 7.41±0.87 | ab | AB |  | 8.39±0.52 | b | B | 20.72 |
| FTLH | 11.39±1.94 | a | A |  | 17.56±0.74 | b | B |  | 15.91±0.50 | b | B | 9.91 |
| STLH | 13.67±2.73 | a | A |  | 24.37±1.78 | b | B |  | 20.69±0.96 | c | B | 8.78 |
| D1 | 4.90±1.30 | a | A |  | 10.15±1.44 | b | B |  | 7.52±0.03 | c | C | -0.07 |
| D2 | 2.29±0.79 | a | A |  | 6.81±1.18 | b | B |  | 4.78±0.69 | c | B | 5.05 |
| SLA | 21.86±2.99 | a | A |  | 36.75±1.84 | b | B |  | 41.17±2.57 | b | B | 40.49 |
| TLA | 25.39±2.87 | a | A |  | 26.30±2.00 | a | A |  | 35.19±6.49 | b | A | 36.16 |
| SPn | 7.41±0.33 | a | A |  | 9.96±0.54 | b | B |  | 9.86±0.98 | b | B | 13.53 |
| TPn | 10.62±0.84 | a | A |  | 12.35±1.25 | a | A |  | 12.39±1.80 | a | A | 7.88 |
| SCi | 247.80±21.81 | a | A |  | 217.52±5.78 | a | A |  | 237.55±14.35 | a | A | 2.10 |
| TCi | 240.15±18.42 | a | A |  | 217.63±7.47 | a | A |  | 235.12±18.37 | a | A | 2.72 |
| SCond | 0.10±0.02 | a | A |  | 0.11±0.01 | a | A |  | 0.13±0.02 | a | A | 23.81 |
| TCond | 0.14±0.04 | a | A |  | 0.14±0.02 | a | A |  | 0.17±0.05 | a | A | 21.43 |
| STr | 3.93±1.57 | a | A |  | 4.01±0.89 | a | A |  | 4.59±0.93 | a | A | 15.62 |
| TTr | 5.05±2.04 | a | A |  | 4.93±1.38 | a | A |  | 5.66±1.76 | a | A | 13.43 |
| Chl a | 0.67±0.03 | a | A |  | 0.83±0.07 | b | AB |  | 0.86±0.06 | b | B | 14.67 |
| Chl b | 0.22±0.01 | a | A |  | 0.28±0.03 | b | B |  | 0.30±0.02 | b | B | 20.00 |
| Car | 0.15±0.01 | a | A |  | 0.19±0.02 | b | B |  | 0.20±0.02 | b | B | 17.65 |
| Chl a/b | 3.09±0.03 | a | A |  | 2.92±0.07 | b | B |  | 2.81±0.03 | c | B | -6.49 |
| Total Chl | 0.89±0.04 | a | A |  | 1.11±0.10 | b | B |  | 1.16±0.08 | b | B | 16.00 |
| PH2 | 75.00±2.50 | a | A |  | 119.33±10.73 | b | B |  | 127.60±16.93 | b | B | 31.32 |
| FBN | 13.33±0.58 | a | A |  | 16.67±0.82 | b | B |  | 17.67±0.58 | b | B | 17.80 |
| BN | 16.33±2.08 | a | A |  | 25.00±4.65 | b | AB |  | 33.00±2.65 | c | B | 59.69 |
| SCY | 87.39±13.10 | a | AB |  | 65.39±19.53 | b | A |  | 107.65±7.73 | a | B | 40.92 |
| LY | 28.94±2.58 | a | A |  | 21.19±6.09 | b | B |  | 35.72±3.05 | c | A | 42.51 |
| BW | 5.29±0.21 | a | A |  | 2.95±0.17 | b | B |  | 3.30±0.06 | c | B | -19.90 |
| SI | 11.54±0.37 | a | A |  | 10.20±1.39 | b | B |  | 12.60±0.49 | c | A | 15.92 |
| LP | 31.15±0.66 | a | A |  | 35.11±1.83 | b | B |  | 33.16±0.56 | ab | AB | 0.09 |
| LI | 5.67±0.10 | a | A |  | 5.11±0.92 | b | A |  | 6.88±0.18 | c | B | 27.64 |
| FL | 28.72±0.23 | a | A |  | 32.81±0.80 | b | B |  | 33.73±1.98 | b | B | 9.64 |
| FS | 28.48±0.72 | a | A |  | 35.35±0.79 | b | B |  | 36.27±1.51 | b | B | 13.65 |
| MIC | 4.81±0.24 | a | A |  | 3.93±0.18 | b | B |  | 3.56±0.07 | c | B | -18.54 |
| FU | 83.59±0.45 | a | A |  | 86.82±1.42 | b | B |  | 85.13±1.19 | a | AB | -0.09 |
| FE | 6.02±0.13 | a | A |  | 7.19±0.30 | b | B |  | 7.06±0.31 | b | B | 6.89 |

**Supplementary Table 3 Statistical analysis of 35 traits phenotypic differences in TM-1 Hai7124 and F1**

**Supplementary Table 4 Haplotype and gene ID of the 10 candidate genes in TM-1 and Hai7124**

| Trait | Haplotype in TM-1 | Number of plants | Haplotype in Hai7124 | Number　of plants | Gene ID in TM-1 | Gene ID in Hai7124 |
| --- | --- | --- | --- | --- | --- | --- |
| BW | CC | 63 | TT | 41 | GH_A10G0723 | GB_A10G0742 |
| CNH | GG | 75 | TT | 57 | GH_A07G0041 | GB_A07G0032 |
| D1 | GA | 63 | AG | 70 | GH_A04G0054 | GB_A04G0055 |
| FLTH | GA | 63 | AG | 70 | GH_A04G0054 | GB_A04G0055 |
| PH1 | TT | 62 | CC | 72 | GH_A13G2361 | GB_A13G2506 |
| PH1 | CA | 66 | TT | 44 | GH_D05G3290 | GB_D05G3544 |
| Sci | CC | 65 | AA | 50 | GH_D04G1426 | GB_D04G1512 |
| Tci | CC | 65 | AA | 50 | GH_D04G1426 | GB_D04G1512 |
| Tcond | CC | 65 | AA | 50 | GH_D04G1426 | GB_D04G1512 |
| LI | TTG | 33 | GCA | 58 | GH_D10G0500 | GB_D06G1730 |
| LP | TTG | 33 | GCA | 58 | GH_D10G0500 | GB_D06G1730 |
| SCY | TTG | 33 | GCA | 58 | GH_D10G0500 | GB_D06G1730 |
| SPn | AA | 53 | GG | 75 | GH_D13G2335 | GB_D13G2423 |
| TPn | GC | 63 | AT | 59 | GH_A01G0707 | GB_A01G0694 |
| TTr | GG | 67 | AA | 74 | GH_A12G0144 | GB_A12G0143 |

| **Supplementary Table 5 Expression of the 10 candidate genes in different tissues of TM-1 and Hai7124** | | | | | | | | | | | | | | | | | | | |
| --- | --- | --- | --- | --- | --- | --- | --- | --- | --- | --- | --- | --- | --- | --- | --- | --- | --- | --- | --- |
| Trait | Gene ID | Root | Stem | Leaf | Petal | Receptacle | Sepal | Epicalyx | Upper of stamen | Bottom half of stamen | Ovule_  0DPA | Ovule_  1DPA | Ovule_  3DPA | Ovule_  5DPA | Ovule_  10DPA | Ovule_  20DPA | Fiber_  10DPA | Fiber_  20DPA | Fiber_  25DPA |
| BW | TM-1_GH_A10G0723 | 0.00 | 0.00 | 0.00 | 0.00 | 0.00 | 0.00 | 0.00 | 0.00 | 0.00 | 0.00 | 0.00 | 0.00 | 0.00 | 0.00 | 0.00 | 0.00 | 0.00 | 0.12 |
|  | Hai7124_GB_A10G0742 | 0.00 | 0.00 | 0.00 | 0.00 | 0.00 | 0.00 | 0.00 | 0.27 | 0.00 | 0.00 | 0.00 | 0.00 | 0.00 | 0.00 | 0.03 | 0.00 | 0.00 | 0.00 |
| CNH | TM-1_GH_A07G0041 | 4.05 | 2.00 | 0.73 | 0.26 | 0.03 | 0.14 | 0.19 | 0.03 | 0.05 | 0.02 | 0.00 | 0.06 | 0.00 | 0.00 | 0.07 | 0.00 | 0.00 | 0.12 |
|  | Hai7124_GB_A07G0032 | 3.91 | 5.55 | 0.93 | 0.54 | 0.42 | 0.73 | 0.60 | 0.07 | 0.00 | 0.00 | 0.00 | 0.02 | 0.00 | 0.00 | 0.65 | 0.00 | 0.00 | 0.23 |
| D1 | TM-1_GH_A04G0054 | 13.51 | 18.08 | 10.75 | 7.98 | 5.32 | 10.00 | 8.13 | 3.80 | 7.17 | 7.48 | 9.36 | 9.79 | 12.20 | 5.76 | 6.75 | 11.47 | 2.44 | 7.17 |
|  | Hai7124_GB_A04G0055 | 13.03 | 12.59 | 8.54 | 4.34 | 5.92 | 5.29 | 5.99 | 2.71 | 6.23 | 7.97 | 9.28 | 7.25 | 8.27 | 7.87 | 10.84 | 3.79 | 3.19 | 8.24 |
| FLTH | TM-1_GH_A04G0054 | 13.51 | 18.08 | 10.75 | 7.98 | 5.32 | 10.00 | 8.13 | 3.80 | 7.17 | 7.48 | 9.36 | 9.79 | 12.20 | 5.76 | 6.75 | 11.47 | 2.44 | 7.17 |
|  | Hai7124_GB_A04G0055 | 13.03 | 12.59 | 8.54 | 4.34 | 5.92 | 5.29 | 5.99 | 2.71 | 6.23 | 7.97 | 9.28 | 7.25 | 8.27 | 7.87 | 10.84 | 3.79 | 3.19 | 8.24 |
| PH1 | TM-1_GH_D05G3290 | 0.93 | 1.85 | 0.98 | 0.00 | 0.10 | 0.27 | 1.72 | 0.38 | 0.76 | 1.25 | 1.28 | 2.13 | 1.62 | 0.15 | 0.58 | 0.32 | 1.04 | 1.04 |
|  | Hai7124_GB_D05G3544 | 0.52 | 1.82 | 1.46 | 0.31 | 0.91 | 0.39 | 0.32 | 0.28 | 1.61 | 1.75 | 2.18 | 1.47 | 1.54 | 1.02 | 0.57 | 1.26 | 0.36 | 0.68 |
| PH1 | TM-1_GH_A13G2361 | 20.14 | 10.53 | 2.00 | 0.34 | 5.98 | 1.10 | 0.15 | 0.03 | 0.01 | 6.85 | 6.15 | 6.28 | 2.84 | 2.12 | 2.34 | 0.69 | 1.53 | 1.42 |
|  | Hai7124_GB_A13G2506 | 11.84 | 8.10 | 1.14 | 0.09 | 9.48 | 1.92 | 0.91 | 0.04 | 0.13 | 5.33 | 4.34 | 6.35 | 6.32 | 1.65 | 3.51 | 0.15 | 0.07 | 3.47 |
| SCi | TM-1_GH_D04G1426 | 10.54 | 4.33 | 44.53 | 0.25 | 0.57 | 4.53 | 1.20 | 1.42 | 0.12 | 0.00 | 0.05 | 0.79 | 1.94 | 5.19 | 0.13 | 4.61 | 0.00 | 1.24 |
|  | Hai7124_GB_D04G1512 | 7.38 | 4.26 | 52.46 | 0.37 | 0.38 | 1.39 | 1.98 | 0.88 | 0.32 | 0.02 | 0.00 | 0.24 | 1.51 | 5.65 | 0.11 | 20.74 | 0.15 | 0.13 |
| Tci | TM-1_GH_D04G1426 | 10.54 | 4.33 | 44.53 | 0.25 | 0.57 | 4.53 | 1.20 | 1.42 | 0.12 | 0.00 | 0.05 | 0.79 | 1.94 | 5.19 | 0.13 | 4.61 | 0.00 | 1.24 |
|  | Hai7124_GB_D04G1512 | 7.38 | 4.26 | 52.46 | 0.37 | 0.38 | 1.39 | 1.98 | 0.88 | 0.32 | 0.02 | 0.00 | 0.24 | 1.51 | 5.65 | 0.11 | 20.74 | 0.15 | 0.13 |
| Tcond | TM-1_GH_D04G1426 | 10.54 | 4.33 | 44.53 | 0.25 | 0.57 | 4.53 | 1.20 | 1.42 | 0.12 | 0.00 | 0.05 | 0.79 | 1.94 | 5.19 | 0.13 | 4.61 | 0.00 | 1.24 |
|  | Hai7124_GB_D04G1512 | 7.38 | 4.26 | 52.46 | 0.37 | 0.38 | 1.39 | 1.98 | 0.88 | 0.32 | 0.02 | 0.00 | 0.24 | 1.51 | 5.65 | 0.11 | 20.74 | 0.15 | 0.13 |
| LI | TM-1_GH_D10G0500 | 9.38 | 7.56 | 1.71 | 6.89 | 8.83 | 8.31 | 5.51 | 6.72 | 8.41 | 3.58 | 4.07 | 9.28 | 8.17 | 11.97 | 4.12 | 16.53 | 32.83 | 20.50 |
|  | Hai7124_GB_D06G1730 | 3.01 | 3.75 | 1.64 | 1.29 | 2.91 | 1.42 | 1.67 | 1.23 | 1.70 | 7.64 | 7.69 | 7.97 | 7.01 | 5.66 | 7.66 | 1.25 | 0.99 | 5.52 |
| LP | TM-1_GH_D10G0500 | 9.38 | 7.56 | 1.71 | 6.89 | 8.83 | 8.31 | 5.51 | 6.72 | 8.41 | 3.58 | 4.07 | 9.28 | 8.17 | 11.97 | 4.12 | 16.53 | 32.83 | 20.50 |
|  | Hai7124_GB_D06G1730 | 3.01 | 3.75 | 1.64 | 1.29 | 2.91 | 1.42 | 1.67 | 1.23 | 1.70 | 7.64 | 7.69 | 7.97 | 7.01 | 5.66 | 7.66 | 1.25 | 0.99 | 5.52 |
| SCY | TM-1_GH_D10G0500 | 9.38 | 7.56 | 1.71 | 6.89 | 8.83 | 8.31 | 5.51 | 6.72 | 8.41 | 3.58 | 4.07 | 9.28 | 8.17 | 11.97 | 4.12 | 16.53 | 32.83 | 20.50 |
|  | Hai7124_GB_D06G1730 | 3.01 | 3.75 | 1.64 | 1.29 | 2.91 | 1.42 | 1.67 | 1.23 | 1.70 | 7.64 | 7.69 | 7.97 | 7.01 | 5.66 | 7.66 | 1.25 | 0.99 | 5.52 |
| SPn | TM-1_GH_D13G2335 | 2.02 | 0.52 | 4.11 | 0.17 | 0.20 | 1.22 | 5.27 | 0.36 | 0.07 | 0.06 | 0.00 | 0.03 | 0.00 | 0.00 | 0.00 | 0.00 | 0.19 | 0.23 |
|  | Hai7124_GB_D13G2423 | 2.15 | 0.26 | 3.76 | 0.07 | 0.10 | 0.73 | 1.05 | 0.03 | 0.00 | 0.00 | 0.02 | 0.00 | 0.06 | 0.00 | 0.00 | 0.00 | 0.00 | 0.00 |
| TPn | TM-1_GH_A01G0707 | 49.10 | 38.06 | 112.72 | 49.64 | 61.32 | 46.47 | 55.90 | 48.07 | 59.91 | 54.40 | 38.99 | 64.17 | 66.56 | 82.38 | 49.37 | 17.91 | 30.06 | 18.63 |
|  | Hai7124_GB_A01G0694 | 50.69 | 41.46 | 68.10 | 49.23 | 56.93 | 52.54 | 59.33 | 46.10 | 50.66 | 44.07 | 41.65 | 43.66 | 26.19 | 41.80 | 59.27 | 65.06 | 58.26 | 54.34 |
| TTr | TM-1_GH_A12G0144 | 17.59 | 17.04 | 34.21 | 15.40 | 26.25 | 18.08 | 18.39 | 14.73 | 11.36 | 24.49 | 21.95 | 13.56 | 15.64 | 17.47 | 11.14 | 6.11 | 11.04 | 10.50 |
|  | Hai7124_GB_A12G0143 | 19.97 | 19.98 | 22.42 | 20.93 | 36.58 | 28.11 | 27.02 | 14.04 | 15.93 | 91.08 | 51.08 | 30.16 | 57.20 | 41.32 | 27.49 | 15.03 | 20.10 | 7.00 |

| Triat | QTL number in At | QTL number in Dt |
| --- | --- | --- |
| Plant type traits at seedling stage | 8 | 4 |
| Leaf morphology and physiological traits at seedling stage | 15 | 22 |
| Leaf chlorophyll content | 5 | 12 |
| Plant type traits at flower and boll stage | 4 | 8 |
| Yield traits | 2 | 12 |
| Fiber quality traits | 7 | 13 |
| Total | 41 | 71 |

**Supplementary Table 6 The distribution of QTLs in the At and Dt subgenomes**
